# The Effect of High Pressure on Polymorphs of a Derivative of Blatter’s Radical: Identification of the Structural Signatures of Subtle Phase Transitions

**DOI:** 10.1021/acs.cgd.2c01422

**Published:** 2023-01-30

**Authors:** Edward T. Broadhurst, Cameron J. G. Wilson, Georgia A. Zissimou, Mayra A. Padrón Gómez, Daniel M. L. Vasconcelos, Christos P. Constantinides, Panayiotis A. Koutentis, Alejandro P. Ayala, Simon Parsons

**Affiliations:** † EaStCHEM School of Chemistry and Centre for Science at Extreme Conditions, The University of Edinburgh, King’s Buildings, West Mains Road, Edinburgh, EH9 3FJ, Scotland; ‡ Department of Chemistry, University of Cyprus, 1678 Nicosia, Cyprus; § Federal University of Ceará, Physics Department, 65455-900, Fortaleza, CE, Brazil; ∥ Department of Natural Sciences, University of Michigan-Dearborn, 4901 Evergreen Road, Dearborn, Michigan 48128-1491, United States

## Abstract

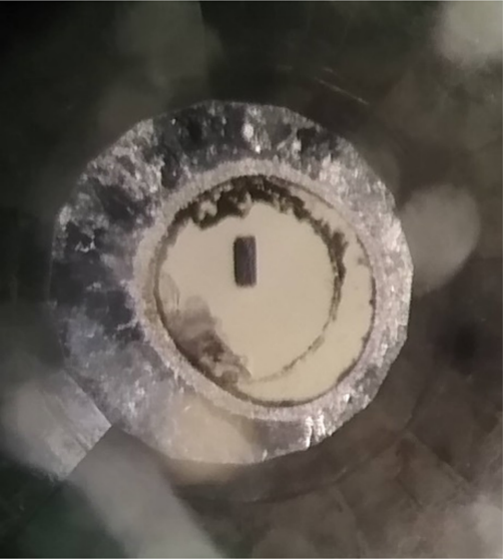

The effect of pressure on the α and β polymorphs of a derivative of Blatter’s radical, 3-phenyl-1-(pyrid-2-yl)-1,4-dihydrobenzo[*e*][1,2,4]triazin-4-yl, has been investigated using single-crystal X-ray diffraction to maximum pressures of 5.76 and 7.42 GPa, respectively. The most compressible crystallographic direction in both structures lies parallel to π-stacking interactions, which semiempirical Pixel calculations indicate are also the strongest interactions present. The mechanism of compression in perpendicular directions is determined by void distributions. Discontinuities in the vibrational frequencies observed in Raman spectra measured between ambient pressure and ∼5.5 GPa show that both polymorphs undergo phase transitions, the α phase at 0.8 GPa and the β phase at 2.1 GPa. The structural signatures of the transitions, which signal the onset of compression of initially more rigid intermolecular contacts, were identified from the trends in the occupied and unoccupied volumes of the unit cell with pressure and in the case of the β phase by deviations from an ideal model of compression defined by Birch–Murnaghan equations of state.

## Abstract 2

Subtle, second order phase transitions occur in the α and β polymorphs of the Blatter radical, 3-phenyl-1-(pyrid-2-yl)-1,4-dihydrobenzo[*e*][1,2,4]triazin-4-yl at pressures of 0.8 and 2.1 GPa.

## 1 Introduction

Although studies of molecular materials at high-pressure have become quite common, there are relatively few structural reports in which the responses of different polymorphs of the same compound are compared.^1^ Organic materials have been studied most extensively, including forms I and II of paracetamol^2^ and the α, β, and γ polymorphs of glycine.^3,4^ A recent study on the orthorhombic and monoclinic polymorphs of histidine revealed first order phase transitions for both.^1^ High pressure has also been used to demonstrate anisotropic compression in two enantiomorphs of 2-(2-oxo-1-pyrrolidinyl)butyramide, which can crystallize in the chirally pure form Levetiracetam or a racemic form, Etiracetam. Raman spectroscopy and an analysis of intermolecular interactions suggest subtle phase transitions in both, at ∼2 and ∼1.5 GPa, respectively.^5^

**Figure 1:**
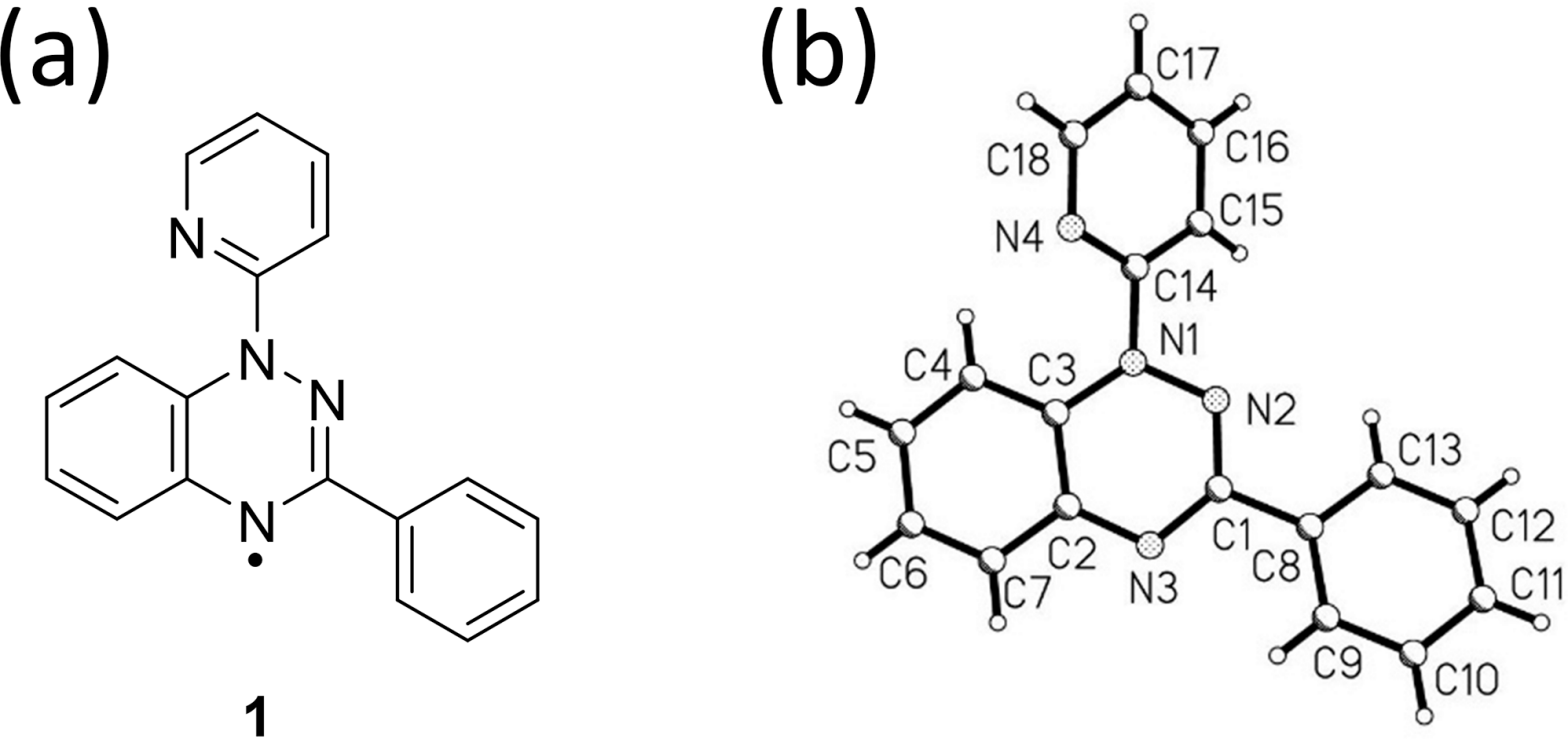
(a) The Blatter’s radical derivative 3-phenyl-1-(pyrid-2-yl)-1,4-dihydrobenzo[*e*][1,2,4]triazin-4-yl (**1**). (b) Diagram depicting the numbering scheme used in this work.

1,3-Diphenyl-1,4-dihydrobenzo[*e*][1,2,4]triazin-4-yl^6^ (Blatter’s radical) and its derivatives are air- and moisture-stable, open-shell species with potential applications as photodetectors,^7,8^ emissive materials for OLEDs,^9^ pH sensors,^10^ liquid crystalline photoconductors,^11−14^ and electroactive building blocks of polymers in purely organic batteries.^15^ The large, delocalized SOMO surface of the benzotriazinyl core in Blatter radicals has many potential sites for intermolecular interactions in crystal structures which, tentatively, can increase the propensity toward polymorphism.^16^ The aim of the present contribution is to compare the effect of pressure on two polymorphs of a derivative of Blatter’s radical, 3-phenyl-1-(pyrid-2-yl)-1,4-dihydrobenzo[*e*][1,2,4]triazin-4-yl (**1**), (Figure 1). The polymorphs are designated **1α**, which is orthorhombic, and **1β**, which is monoclinic. Both forms are shown to undergo second order phase transitions at high pressure, and we describe below the extent to which a structural signature for this class of subtle transformation can be identified.

## 2 Experimental Section

### 2.1 Single Crystal X-ray Diffraction

The Blatter’s radical derivative, 3-phenyl-1-(pyrid-2-yl)-1,4-dihydrobenzo[*e*][1,2,4]triazin-4-yl (**1**) was prepared according to the procedure in ref (17). Single crystals suitable for X-ray diffraction were obtained from a supersaturated solution of **1** in *n*-hexane. Polymorph **2β** was obtained initially from the hot solution as it cooled to room temperature. Polymorph **2α** then formed slowly when the same solution was allowed to stand at room temperature.^16^

Diffraction data were measured on a Bruker D8 Venture diffractometer using Mo Kα radiation (λ = 0.71073 Å) at pressures up to 5.76 GPa for **1α** and 7.42 GPa for **1β**, across two separate crystal loadings for each. The pressure limits were defined by the loss of Bragg diffraction, suggesting the onset of amorphization. The crystals were loaded into a Merrill–Bassett diamond-anvil cell (DAC) with half opening angle of 38°, 600 μm Boehler-Almax diamonds, and tungsten carbide backing plates.^18^ A tungsten gasket of thickness 300 μm indented to 155 μm and hole diameter of 300 μm was used, along with a 4:1 mixture of methanol and ethanol as a pressure-transmitting medium.^19^ A small ruby chip was also included in the sample loading and the ruby fluorescence method was used to measure the pressure.^20^ The standard deviation of all pressures quoted is 0.05 GPa.

Data were collected as in ref (21) and processed using the APEX3 software package.^22^ The initial structures were solved using dual-space methods [SHELXT],^23^ and refinements at higher pressure started from the atomic coordinates of the preceding pressure point. Refinement was by full-matrix least-squares on |*F*|^2^ (SHELXL, from within the OLEX2 graphical user interface).^24,25^ Intramolecular bond distances and angles in all refinements against data collected at high pressure were restrained to those observed at ambient pressure. Where possible, non-hydrogen atoms were refined with anisotropic displacement parameters subject to enhanced rigid-bond restraints.^26^ Crystals of **1β** diffracted more weakly than those of **1α**, leading to poorer data quality statistics and lower precision in the refined parameters; in some cases it was necessary to mix isotropic and anisotropic refinement for different atoms in the same structure. Nevertheless, the data do establish consistent trends in volumes and contact distances with pressure. Hydrogen atoms were placed in calculated positions and constrained to ride on their parent atoms. Crystal and refinement data for both **1α** and **1β** are listed in Tables S1 and S2 in the Supporting Information.

### 2.2 Raman Spectroscopy

Raman spectra were acquired on a LabRAM HR (Jobin-Yvon HORIBA) spectrometer, which is an 800 mm focal length Czerny-Turner type spectrograph (spectral resolution ∼1 cm^–1^) equipped with a grating with 1800 grooves/mm and a charge-coupled device (CCD) cooled with liquid nitrogen. An argon laser operating at a wavelength of 488 nm was used for the excitation of the samples. The laser beam was focused on the sample surface using a microscope (OLYMPUS) with a long-working distance 50× lens with a numerical aperture of 0.75, forming a spot of approximately 4 μm on the surface of the sample. High pressure was applied to the samples using a diamond anvil cell (DAC). To avoid sample decomposition under laser irradiation, MeOH/EtOH was replaced as a pressure-transmitting medium by nujol. Nujol is a mineral oil (CAS number 8012–95–1), which provides quasi-hydrostatic conditions to 7–10 GPa,^27^ confirmed in our measurements by the symmetrical ruby emission bands observed at all pressures. Pressures were measured using the ruby fluorescence method.^20^ The maximum pressures reached were 5.36 GPa for **1α** and 5.43 GPa for **1β**. The deconvolution of the recorded spectra into a set of Lorentzian line profiles was performed using the Fityk software.^28^

### 2.3 Calculation of Intermolecular Interaction Energies via the PIXEL Method

Lattice energies (Figure S2) and intermolecular interaction energies were calculated using the semiempirical PIXEL method^29−31^ using the CLP-PIXEL suite through the MrPIXEL interface.^32^ The energies were evaluated within a cluster of radius 14 Å with electron densities obtained on a grid of 0.08 × 0.08 × 0.08 Å^3^ in DFT calculations using the 6-31G** basis set and the B3LYP level of theory in Gaussian09.^33^ The condensation level was 4.

### 2.4 Other Programs Used

Structures were visualized in Mercury and DIAMOND.^34,35^ The principal axes of strain were calculated using STRAIN^36^ and EoSFit7-GUI^37^ was used for equation of state fitting. The void space analyses were carried out with Mercury and CellVol.^38^

## 3 Results and Discussion

### 3.1 Comparison of the Two Polymorphs at Ambient Conditions

3-Phenyl-1-(pyrid-2-yl)-1,4-dihydrobenzo[*e*][1,2,4]triazin-4-yl (**1**) (Figure 1a) consists of a central benzo-1,2,4-triazinyl unit with a phenyl ring attached to C1 (referred to below as C-Ph) and a pyridyl group attached to N1 (N-Pyr). Polymorph **1α** crystallizes in the orthorhombic space group *P*2_1_2_1_2_1_, while **1β** is monoclinic, space group *P*2_1_/*c*. Both forms contain one molecule in the asymmetric unit. The atom-numbering scheme is presented in Figure 1b.

The intramolecular bond distances and angles are the same in each polymorph, but there are small differences in the orientations of the substituents (Figure S1 in the Supporting Information). The N-Pyr torsional angle (C3–N1–C14–N4) is 39.0(3)° and 37.9(5)°, while the C-Ph torsional angle (N3–C1–C8–C9) is −15.6(3)° in **1α** and −1.6(5)° in **1β**, respectively. The triazinyl planar ring “hinge” angle, described by the mean planes of N1–N2–C1–N3 and N1–C3–C2–N3, is 7.4(1)° for **1α** and 4.5(2)° for **1β**, conferring greater planarity in **1β**. The lattice energies at ambient conditions, calculated using the PIXEL method, are −138.8 and −136.6 kJ mol^–1^ for **1α** and **1β** (Figure S2), respectively. **1α** is therefore the thermodynamically stable form, but its density (1.340 g cm^–3^) is also lower than **1β** (1.376 g cm^–3^).

**Figure 2:**
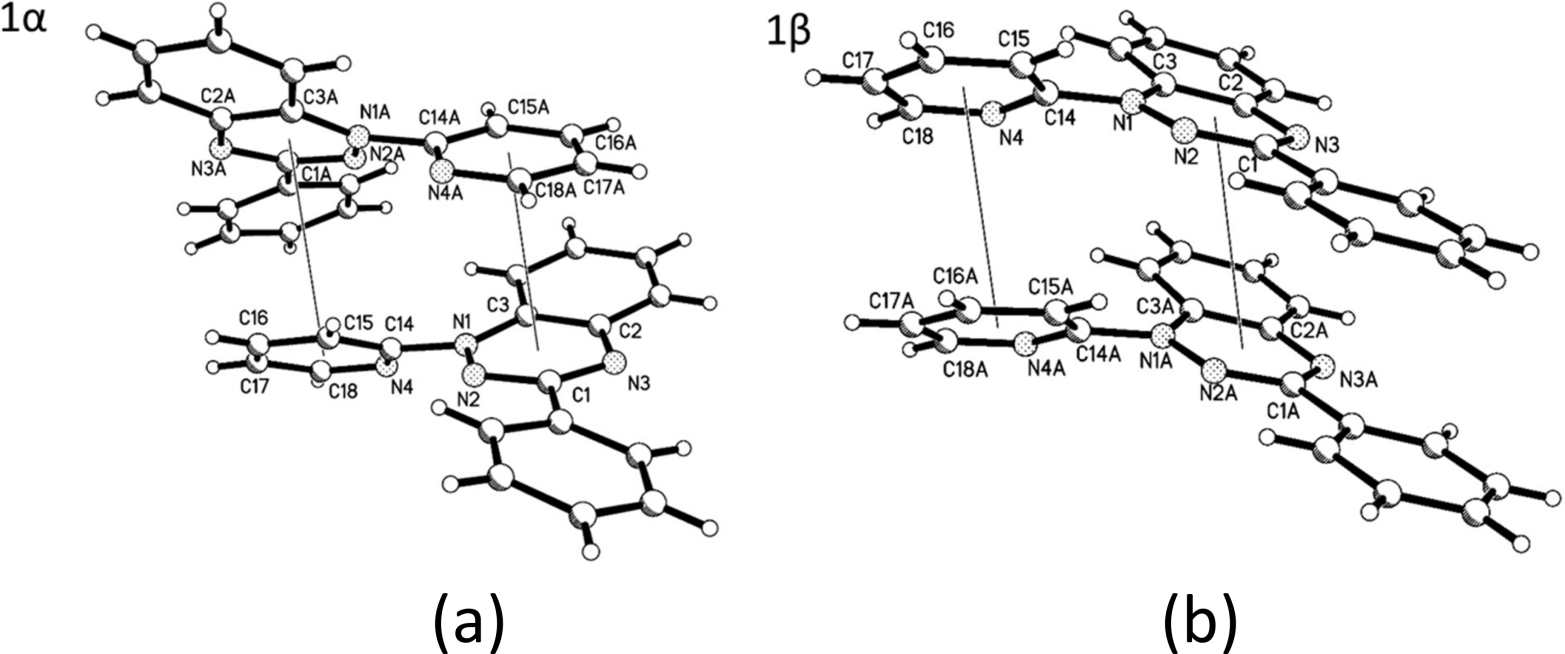
Formation of the different π-stacking interactions via a 2_1_ screw in **1α** and lattice translation in **1β**.

Intermolecular interaction energies, evaluated by the Pixel method, are listed in Tables 1 (**1α**) and 2 (**1β**). Symmetry equivalent interactions are labeled A and A′, *etc.* The strongest interaction in both structures occurs through stacking of the molecules (interaction A in Tables 1 and 2, – 55.6 kJ mol^–1^ in **1α** and −49.5 kJ mol^–1^ in **1β**). In polymorph **1α** the stacking is generated by a 2_1_ axis along **a**, while in **1β** it is generated through lattice repeats along **b**. As a result, in **1α** the π-stacking interactions are between the benzotriazinyl and the N-Pyr moieties (Figure 2a) in which the ring-centroid distances are 3.5375(14) and 4.0076(14) Å. In polymorph **1β** the stacking occurs through triazinyl–triazinyl and pyridyl–pyridyl interactions (Figure 2b), both measuring 3.8612(19) Å, which is similar to the average of the two stacking distances seen in **1α**.

**Table 1:** Intermolecular Interaction Energies in the First Coordination Sphere for **1α**^a^

Interaction label	Symmetry	Centroid distance (Å)	Coulombic	Polarization	Dispersion	Repulsion	Total
A	–^1^/_2_+*x*, ^1^/_2_–*y*, 1–*z*	4.746	–16.7	–6.1	–74.0	41.2	–55.6
B	1–*x*, –^1^/_2_+*y*, ^3^/_2_–*z*	8.938	–6.4	–4.0	–29.1	16.8	–22.6
C	^3^/_2_–*x*, 1–*y*, –^1^/_2_+*z*	9.969	–7.7	–3.1	–21.2	15.0	–17.1
D	–^1^/_2_+*x*, ^3^/_2_–*y*, 1–*z*	9.442	–3.6	–1.5	–12.1	5.0	–12.2
E	^1^/_2_–*x*, 1–*y*, –^1^/_2_+*z*	10.033	–3.4	–1.4	–14.1	7.1	–11.9
F	*x*, –1+*y*, *z*	10.996	–0.9	–1.8	–8.8	6.0	–5.4
G	1–*x*, ^1^/_2_+*y*, ^1^/_2_–*z*	11.906	–0.7	–0.8	–5.9	2.4	–5.0
H	^3^/_2_–*x*, –*y*, –^1^/_2_+*z*	12.396	–1.0	–0.5	–4.4	1.5	–4.4

a All energies are in kJ mol^–1^. Operators for equivalent contacts are A′: ^1^/_2_+*x*, ^1^/_2_–*y*, 1–*z*; B′: 1–*x*, ^1^/_2_+*y*, ^3^/_2_–*z*; C′: ^3^/_2_–*x*, 1–*y*, ^1^/_2_+*z*; D′: ^1^/_2_+*x*, ^3^/_2_–*y*, 1–*z*; E′: ^1^/_2_–*x*, 1–*y*, ^1^/_2_+*z*; F′: *x*, 1+*y*, z; G′: 1–*x*, – ^1^/_2_+*y*, ^1^/_2_–*z*; H′: ^3^/_2_–*x*, – *y*, ^1^/_2_+*z*.

**Table 2:** Intermolecular Interaction Energies in the First Coordination Sphere for **1β**^a^

Interaction label	Symmetry	Centroid distance (Å)	Coulombic	Polarization	Dispersion	Repulsion	Total
A	*x*, –1+*y*, *z*	3.861	–3.3	–5.3	–80.0	39.2	–49.5
B	2–*x*, –^1^/_2_+*y*, ^3^/_2_–*z*	9.566	–6.5	–3.7	–26.0	15.8	–20.4
C	*x*, ^1^/_2_–*y*, ^1^/_2_+*z*	10.091	–10.1	–3.8	–20.8	14.6	–20.1
D	1–*x*, 1–*y*, 1–*z*	9.020	–0.8	–3.4	–25.5	14.5	–15.2
E	*x*, ^3^/_2_–*y*, –^1^/_2_+*z*	10.065	–3.1	–1.6	–14.0	6.5	–12.2
F	1–*x*, 2–*y*, 1–*z*	9.786	–4.0	–1.2	–13.6	6.7	–12.0
G	1–*x*, –^1^/_2_+*y*, ^1^/_2_–*z*	12.895	–1.7	–0.8	–8.8	4.7	–6.7
H	2–*x*, 1–*y*, 1–*z*	11.730	–0.7	–1.9	–11.0	7.5	–6.0

a All energies are in kJ mol^–1^. Operators for equivalent contacts are A′: *x*, 1+*y*, *z*; B′: 2–*x*, ^1^/_2_+*y*, ^3^/_2_–*z*; C′: *x*, ^1^/_2_–*y*, – ^1^/_2_+*z*; E′: *x*, ^3^/_2_–*y*, ^1^/_2_+*z*; G′: 1–*x*, ^1^/_2_+*y*, ^1^/_2_–*z*.

**Figure 3:**
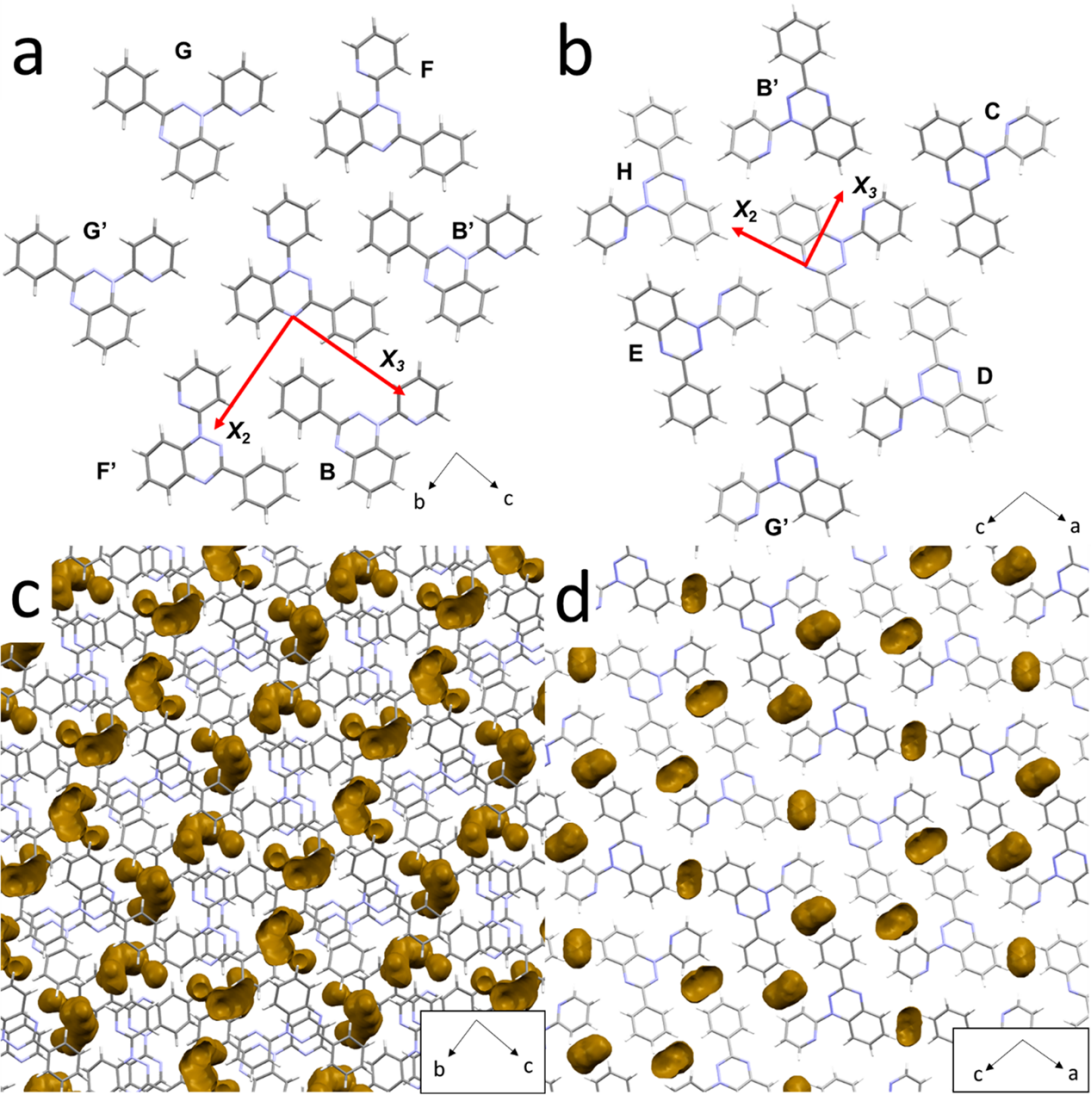
Strain axes *X*_2_ and *X*_3_ viewed along the largest eigenvalue *X*_1_ in (a) polymorph **1α** and (b) polymorph **1β**. Molecules are labeled as in Tables 1 and 2. Void distributions visualized using Mercury with a probe radius of 0.75 Å and a grid spacing of 0.3 Å are shown in (c) for polymorph **1α** and (d) polymorph **1β**. The distribution of the voids in **1α** (b) is more uniform, whereas the voids are aligned approximately in the direction of *X*_2_ in **1β** (d).

The stacks are arranged so that when **1α** is viewed along **a** and **1β** is viewed along **b**, each stack is surrounded by six others. In **1α** a cross-section through the unit cell perpendicular to the stacks reveals layers parallel to the *bc* planes containing interactions B/B′, F/F′, and G/G′ (Figure 3a). Interaction B (−22.6 kJ mol^–1^) contains a contact between N3 and H12 (2.78 Å), while F and G are best regarded as nonspecific whole-molecule dispersion interactions. The other interactions in Table 1 are formed “diagonally” between stacks and layers, interaction C featuring a short N···H contact (N4···H10 = 2.69 Å). Layers are also formed perpendicular to the stacks in polymorph **1β**, but they are much more corrugated (Figure S3) than in the α phase. Interactions B′, D, E, C, G′, and H form within the layers (Figure 3b), B and C, respectively, containing N4···H5 (2.70 Å, 160°) and N3···H17 (2.64 Å, 167°) contacts.

### 3.2 Response of the Unit Cell Dimensions to Pressure

The variation of unit cell volume with pressure is shown for both polymorphs in Figure S4. No discontinuities are seen for either phase in the volume or in the lattice energies (Figure S2). The bulk moduli, determined from fitting third order Birch–Murnaghan equations of state (EoS) with volumes (*V*_0_) fixed to the values measured at ambient conditions, are *K*_0_ = 7.7(4) GPa with *K*_0_′ = 9.5(8) for **1α**, and *K*_0_ = 7.5(5) GPa with *K*_0_′ = 9.8(9) for **1β**. The bulk moduli of the two polymorphs are the same within error, despite the higher density of **1β**, and similar to those of the Blatter’s radical itself [7.4(6) GPa and 9.33(11)]^39^ and other molecular solids such as C_6_Br_6_ (*K*_0_ = 9.07 GPa), anthracene (7.5 GPa), and hexamethylbenzene (7.2 GPa).^40^ Animations showing the path of compression of each polymorph are available in the SI (Supplementary Movies 1–3 for **1α** and 4–6 for **1β**).

**Figure 4:**
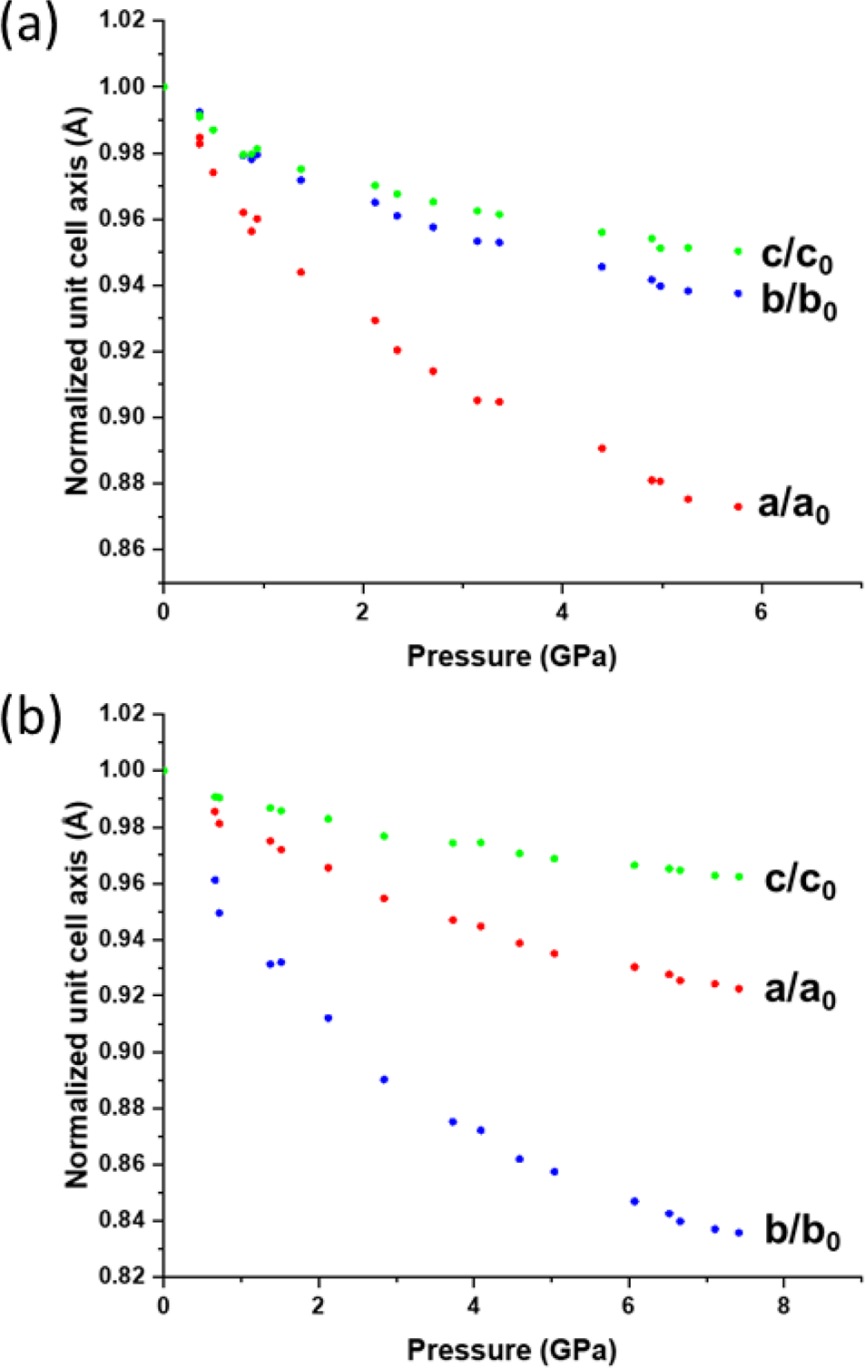
Change in unit cell dimensions for (a) **1α** and (b) **1β** across the pressure series.

The compression is anisotropic (Figure 4). The direction of highest compression aligns with the strongest π-stacking interactions for both structures, the *a* axis compressing by 13% for polymorph **1α** and *b* axis by 17% for **1β**. The changes in the *b* and *c* directions are similar for **1α** at 6% and 5%, respectively, while the *a* axis changes by 8% and the *c* axis by 4% in **1β**. In polymorph **1β** the β angle decreases by 1.5%, causing sin β to increase over the pressure series (Figure S5), adding a positive rather than negative contribution to the volume change.

The principal axes of strain are parallel to the *a*, *b*, and *c* axes in **1α** with eigenvalues that follow the same numerical order as the changes in the cell dimensions themselves (Table 3, with values determined from the structure at 4.98(5) GPa), the largest, *X*_1_, being parallel to *a*, and the other two axes, *X*_2_ and *X*_3_, having quite similar values.

**Table 3:** Principal Axes of Strain in **1α** and **1β** at 4.98 and 5.04 GPa, Respectively^36^

polymorph	axes	eigenvector	eigenvalue
**1α**	*X*_1_	[100]	–0.11930(18)
	*X*_2_	[010]	–0.0602(2)
	*X*_3_	[001]	–0.0489(4)
**1β**	*X*_1_	[010]	–0.14264(16)
	*X*_2_	[0.049, 0, −0.004]	–0.0655(2)
	*X*_3_	[0.026, 0, 0.056]	–0.0211(3)

In **1β**, symmetry requires that one axis of strain is parallel to the *b* axis. Consistent with the trends in the cell dimensions, this corresponds to the largest eigenvalue, which has a similar value to that seen at a similar pressure in **1α** (Table 3, values determined from the structure at 5.04(5) GPa). In contrast to **1α**, the two smaller eigenvalues are quite different from one another, reflecting the differences between the trends in the cell dimensions shown in Figure 4.

The origin of the difference may lie in the distribution of voids (Figure 3c and d). In **1α** the voids are quite uniformly distributed around the stacks, while those in **1β** can be considered as aligning in the along the direction of *X*_2_, with a lower density of voids along *X*_3_, promoting a more anisotropic path of compression. These differences are explored in more detail below. GIF animations of compression of the voids between the stacks are available in the SI (Supplementary Movies 7 and 8).

### 3.3 High Pressure Raman Spectroscopy

Since polymorphs **1α** and **1β** quickly decompose under laser excitation, Raman spectra were recorded using very low laser power. The spectra at ambient conditions are shown in Figure S6. Several features distinguish the polymorphs, such as the bands related to the CH bending (1420 to 1530 cm^–1^) and double bonds (1530 to 1660 cm^–1^). Elsewhere, bands between 300 and 1100 cm^–1^ are at similar wavenumbers despite minor relative intensity changes.

**Figure 5:**
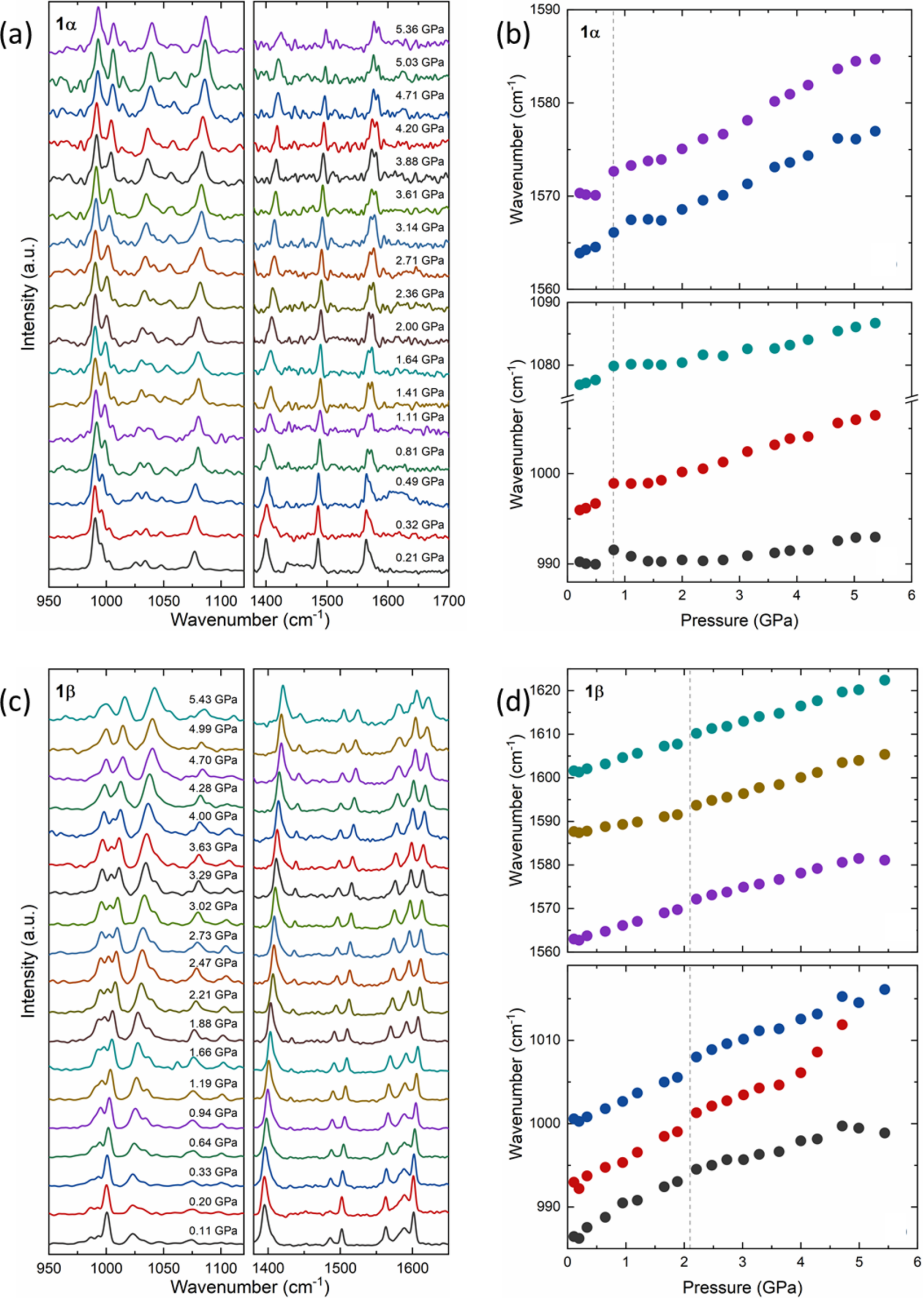
(a) Raman spectra of **1α** as a function of pressure. (b) Trends with respect to pressure in the wavenumbers of modes near 1600 and 1000 cm^–1^. (c) and (d) show analogous plots for **1β**.

The main effect of hydrostatic pressure is the hardening of the vibrational modes, which is consistent with the unit cell volume reduction observed in the X-ray diffraction experiments (Figure 5). No apparent structural changes can be identified by directly examining the spectra since no new bands are observed. The spectra become gradually broader and weaker and cannot be resolved above ∼5.5 GPa. This process is consistent with the trend toward an amorphization observed in the diffraction experiments. The difference in the amorphization pressures of 5.36 GPa (**1β**) and 5.43 GPa (**1β**) from those seen in the diffraction experiments (above 5.76 and 7.42 GPa, respectively) is probably a reflection of the differing hydrostatic properties of the pressure-transmitting media, nujol and 4:1 methanol–ethanol in the two experiments. The vibrational spectra are recovered upon releasing the pressure (Figure S7) indicating that the amorphization is reversible.

A more detailed analysis of the Raman spectra is enabled by deconvolution of the bands into a set of Lorentzian profiles. As the pressure dependence of the Raman spectra is quite subtle, the analysis will focus on one region where there is little difference between the polymorphs (∼1000 cm^–1^) and another where these forms can be fingerprinted (∼1600 cm^–1^). The same effects are seen in other spectral regions.

The pressure dependence of selected bands of **1α** is shown in Figure 5b. There is a clear discontinuity around 0.8 GPa; the same discontinuity is observed in all bands and is reversible, as verified in the spectra recorded after pressure release. In the case of form **1β**, there is also a discontinuity in the wavenumber pressure dependence of the Raman bands (Figure 5d) around 2.1 GPa. The hardening of the bands is accompanied by their broadening, which leads to the merging of some bands, exemplified in Figure 5d around 1015 cm^–1^ at 5 GPa. This behavior is consistent with a trend toward amorphization.

The discontinuities that occur in the Raman spectra for **1α** at 0.8 GPa and **1β** at 2.1 GPa are indicative of phase transitions and appear to be inconsistent with the smooth, featureless trends seen in the unit cell dimensions and volume with pressure. However, the Raman anomalies in both sets of spectra occur in all modes but without the emergence of any new signals, implying that they are related to a change in compressibility rather than a sudden modification of the molecular conformation or packing arrangement. This conclusion can be explored further in a more detailed analysis of the variation of the unit cell volumes.

### 3.4 Volume Analysis of the Phase Transitions

The volume of a crystal structure can be partitioned into regions occupied by molecules and their network of intra- and intermolecular interactions and unoccupied interstitial voids. We refer to these as the “network” and “void” regions, respectively, and their volumes are *V*_net_ and *V*_void_; the structural signature of a high-pressure phase transition can be clearer in these quantities than in the overall unit cell volume or unit cell dimensions.^38^

Attempts to fit *V*_net_ and *V*_void_ to third order Birch–Murnaghan and Vinet equations of state respectively are shown for each polymorph in Figures S8 and S9 in the Supporting Information, with numerical results presented in Table 4. The trends in the void volumes are smooth for both and adequately fitted to single equations of state with χ^2^ values near unity.

**Table 4:** Values Determined from the EoS Fitted for the Total (*V*_tot_), Network (*V*_net_), and Void (*V*_void_) Volumes, Respectively, For Each Polymorph Across the Entire Pressure Range Applied in Each Study^a^

		*V*_0_ (Å^3^)	*K*_0_ (GPa)	*K*_0_′	χ^2^
	*V*_tot_	1414.44	7.7(4)	9.5(8)	1.01
**1α**	*V*_net_	1070.81	95(3)	–2(1)	1.63
	*V*_void_	343.63	2.2(1)	1.1(1)	1.13
	*V*_tot_	1377.30	7.5(5)	9.8(9)	1.50
**1β**	*V*_net_	1068.51	106(6)	–3(1)	7.21
	*V*_void_	308.79	2.1(1)	0.6(6)	1.08

a *V*_tot_ = *V*_net_ + *V*_void_.

**Figure 6:**
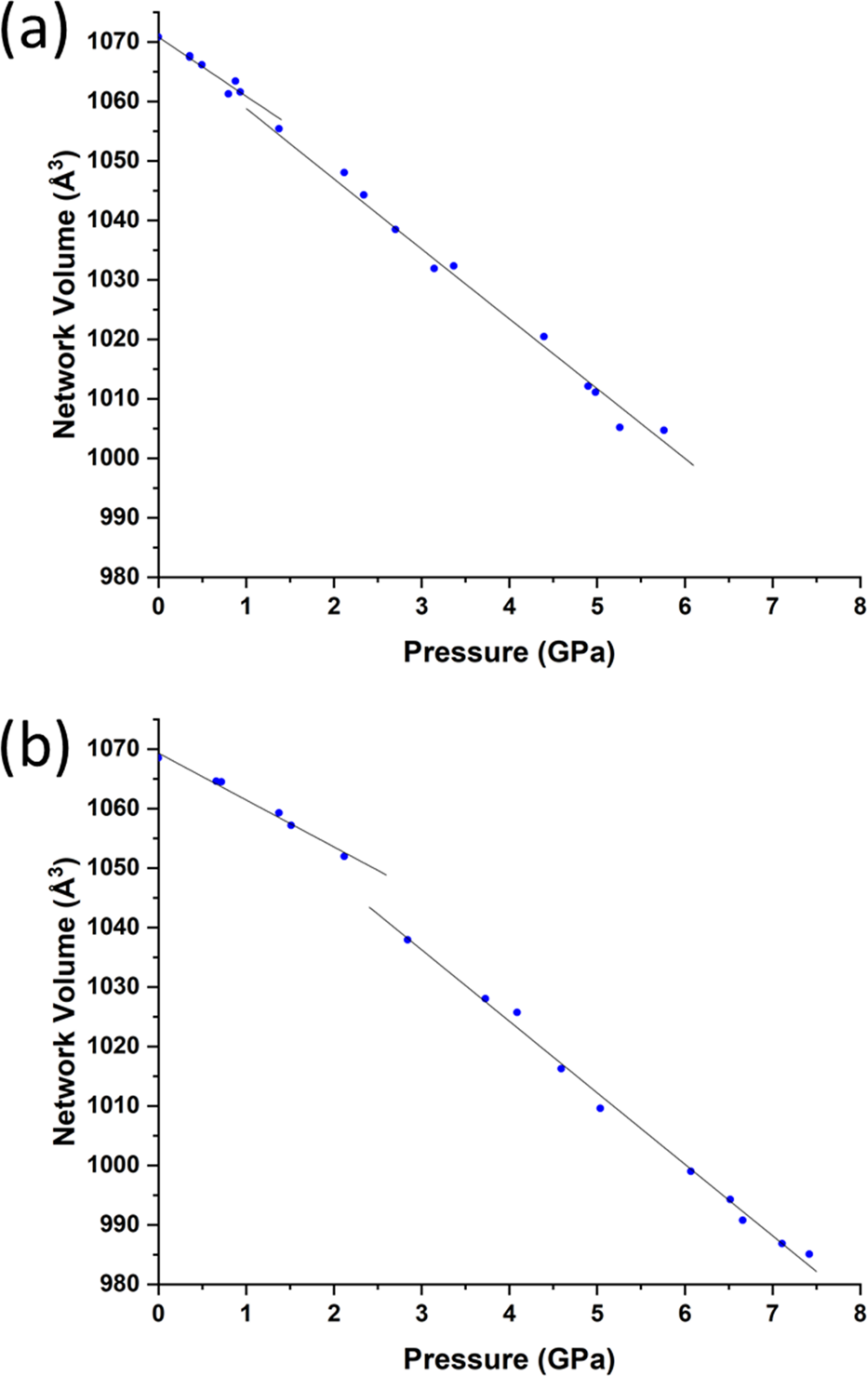
(a) Linear fits to the variation of the network volume with pressure of **1α** above and below 0.8 GPa. (b) Analogous fits for the **1β** phase above and below 2.12 GPa. Error bars lie within the symbols.

Although an acceptable fit to a single equation of state across the full pressure range can also be obtained for the network volume of **1α**, it is possible to discern that the trend can also be fitted to two straight lines, obtained using Murnaghan equations of state with *K′* fixed to −1, above and below 0.8 GPa (Figure 6a). The network bulk moduli before and after the transition are 107(8) and 87(3) GPa (χ^2^ = 0.75 and 1.98, respectively).

A similar, but much clearer, discontinuity occurs in the network volume of **1β**, which consists of two separate linear regions (Figure 6b) above and below 2.12 GPa with bulk moduli of 144(5) and 94(1) GPa, respectively. These fits, with χ^2^ = 1.17 and 2.42, are superior to a fit to a single equation of state (χ^2^ = 7.21).

The discontinuities suggest, in agreement with the Raman data, that the phase transitions occurring in **1α** at 0.8 GPa and in **1β** above 2.1 GPa are related to a switch in which the mechanism of compression shifts toward the network of intermolecular interactions. The discontinuity in compressibility rather than the unit cell volume itself implies that both transitions are second order.

### 3.5 Effect of Pressure on Intramolecular Geometry

The molecular volume for polymorph **1α** at ambient pressure is 268.2(2) Å^3^ and at 5.76 GPa is 267.6(3) Å^3^. The molecular volume for **1β** at ambient pressure is 267.8(2) Å^3^ and at 7.42 GPa is 267.6(3) Å^3^. The small magnitude of these differences indicates that most of the reduction in the network volume in both polymorphs is due to intermolecular compression.

In Blatter’s radical itself,^39^ a first order phase transition at 5.34 GPa was driven by the onset of rotation of the N-phenyl rings. The ring substituents in polymorphs **1α** and **1β** rotate in a similar fashion with increasing pressure, but the rotation is continuous. The N-Pyr torsion in **1α** decreases from 39.0(3)° at ambient to 32.1(7)° at 4.40 GPa and then increases to 36(3)° at 5.76 GPa. In **1β**, the angle changes from 37.9(5)° to 28.7(7)° from ambient to 7.42 GPa, with no discontinuity. The C-Ph torsion angle changes from −13.8(3)° to −16(4)° between ambient and 5.76 GPa in **1α** and from −1.6(2)° to −1.5(4)° in **1β**. Plots of each torsion angle with pressure are available in the SI (Figure S10).

The triazinyl moiety, where the π-stacking interaction forms, is sensitive to changes in planarity and modifications have been shown to affect the location and magnitude of the spin density.^16,41^ The hinge angle (calculated between the mean planes N1–N2–C1–N3 and N1–C3–C2–N3) shows distinct and opposite reactions to pressure increase, **1α** exhibits an angle increase by 5.98°, while in **1β** it decreases by 2.13° (Figure S11).

The continuous changes in the intramolecular bonding parameters imply that there is little to suggest that there is any intramolecular feature associated with the second order transition in either polymorph.

### 3.6 Effect of Pressure on Intermolecular Contact Distances

**Figure 7:**
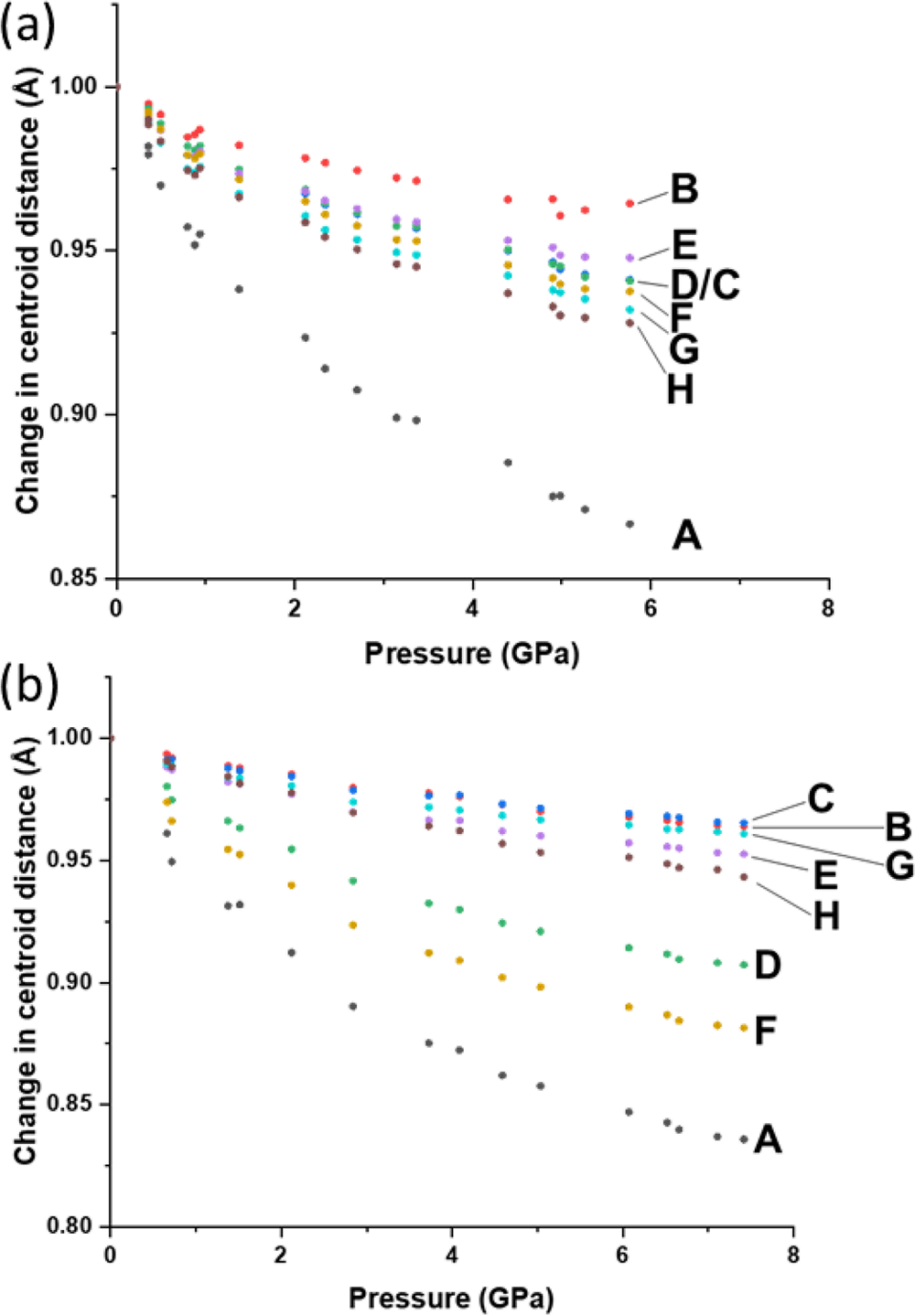
Relative change to the centroid distance for each interaction with pressure for (a) **1α** and (b) **1β**.

Centroid–centroid distances for each interaction in Tables 1 and 2 are plotted against pressure in Figure 7. In polymorph **1α**, the centroid distance of interaction A compresses the most, reducing by 14%. This contrasts with the other interactions which all show very similar but markedly less compression. This behavior can be linked to the uniform distribution of the voids in the structure, as also noted above. The *X*_2_ and *X*_3_ strain tensors are reflected in the centroid distances, where *X*_2_ mostly compressed toward interaction F and *X*_3_ compresses the least, owing proximity to interaction B. Interaction B has molecules which are almost coplanar with short H···H, C···H, and N···H contacts between C-Ph groups (Figure S12).

**Figure 8:**
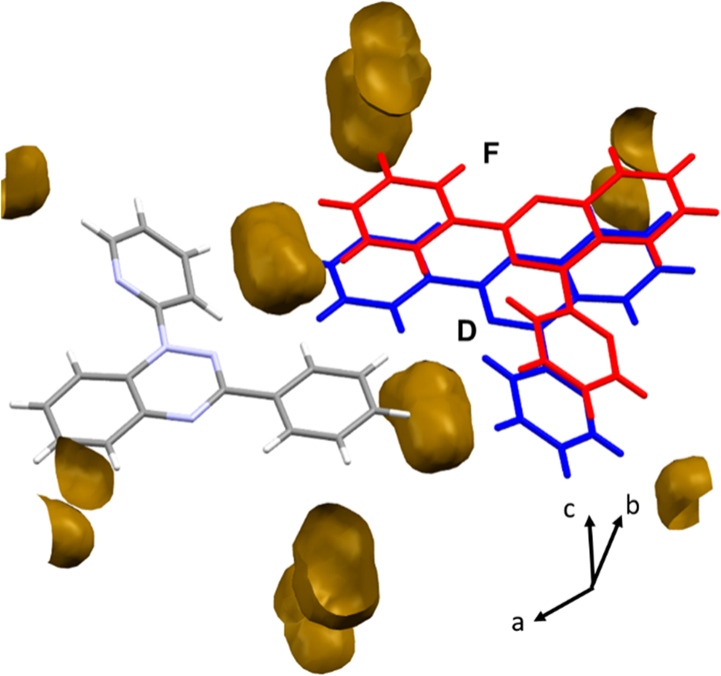
Voids (also shown in Figure 3d) in the regions between the gray reference molecule and the blue and red molecules in interactions D and F, respectively.

The stacking interaction (A) is also the most compressible in polymorph **1β**, decreasing by 16.5% over the pressure series. By contrast with **1α**, there are marked differences between the other interactions, with D and F compressing substantially more than the others. The distribution of voids allows the compression of C-Ph and N-Pyr toward one another in interaction D and F (Figure 8). The largest strain tensor component *X*_1_ is aligned with the compression along interaction A, while *X*_2_ is aligned in the direction of compression of both interactions F and D. The differences between the strain tensor components are thus seen to reflect the changes in intermolecular interaction distances with pressure, with the greater anisotropy in **1β** identified with the high compressibility of interactions D and F.

The linear modulus (*M*_0_) of each interaction was calculated using third order Birch–Murnaghan EoSs fitted to the centroid distances from the entire pressure series for **1α** (Figures S13–S20) but only those up to 2.12 GPa for **1β** (Figures S21–S28). The results are in Table 5. As with a volume-based bulk modulus, a high linear modulus is associated with an incompressible interaction, while deviations from the trend expressed by an EoS can be associated with nonideal behavior or a phase transition.

**Table 5:** Estimation for Linear Modulus (*M*_0_) of Each Interaction in Each Polymorph^a^

	**1α**			**1β**		
Interaction	*M*_0_	*M*_0_′	χ^2^	*M*_0_	*M*_0_′	χ^2^
A	14(1)	15(1)	0.60	11(1)	14(1)	1.02
B	39(9)	106(37)	4.53	96(6)	46(6)	1.10
C	30(2)	49(6)	1.08	64(7)	100(20)	1.42
D	38(2)	35(4)	0.94	24(2)	25(2)	1.08
E	18(3)	120(21)	1.01	39(4)	86(13)	1.25
F	29(2)	41(5)	0.93	17(1)	19(1)	1.31
G	19(2)	62(8)	0.82	31(6)	180(40)	1.33
H	22(2)	40(4)	0.98	66(4)	24(3)	2.36

a Centroid distances from the entire pressure series were used for **1α** and only the centroid distances up to 2.12 GPa were used in **1β**. All were fitted using a third order Birch-Murnaghan EoS.

Attempts to fit the data for **1β** shown in Figures S21–S28 to single equations of state led to poor fits with high values of χ^2^. This demonstrates that although the effects of the transition are quite subtle and difficult to observe in the trends in cell parameters or from obvious discontinuities in intermolecular interactions distances, they can be seen in deviations from ideal behavior in the contact distances, as well as in the network volume as described above. Beyond the phase transition, interactions B, C, E, G, and H, and to a lesser extent F, compress more than would be anticipated in the compression seen up to 2.12 GPa (all the observed points lie below the EoS line). The already soft interactions A and D compress ideally, while for F the deviation from ideality is relatively modest. The phase transition in **1β** is thus associated with a general softening of the more incompressible contacts, rather than a step change in any one contact.

The phase transition in polymorph **1α** occurs at only 0.8 GPa, and as shown in Figure 6a, it is difficult to establish a distinctive equation of state in such a limited pressure range. The transition is also structurally more subtle than in **1β**. As a result, all interactions in polymorph **1α** appear to conform to ideal behavior across the entire pressure range studied.

## 4 Conclusions

The crystal structures of the 3-phenyl-1-(pyrid-2-yl)-1,4-dihydrobenzo[*e*][1,2,4]triazin-4-yl polymorphs, **1α** and **1β**, have been investigated up to 5.76 and 7.42 GPa, respectively. The strongest contacts in both polymorphs are π-stacking interactions, but the crystallographic axes along which they are distributed experience the most compression at elevated pressure. Perpendicular to this direction, the compression is governed by the distribution of interstitial voids.

As is the case with other molecular structures, the path of compression in both polymorphs involves reduction of the void space. This is very clearly illustrated by the difference in the bulk moduli of the void volume, in the region of 2 GPa for both phases, compared to the occupied “network” volume, ∼100 GPa in both cases, similar to the total bulk modulus of a first-row transition metal.

Although no first order phase transitions occur for either polymorph, there is a very clear discontinuity in the vibrational frequencies and network volume of **1β** above 2.1 GPa. Analysis of the intermolecular contact distances shows that this change is associated with a shift in the mechanism of compression toward the initially less compressible intermolecular contacts. Specific contacts could be identified by their deviations from an ideal model of compression expressed in a linear third order Birch–Murnaghan equation of state. The term “linear” here refers to the variation of distance with pressure instead of volume, rather than indicting that the equation of state follows a straight line.

The situation is more ambiguous for **1α**. The Raman data show a very clear anomaly at 0.8 GPa, but corresponding discontinuity in the network volume is very slight and, in all candor, was not detected until the Raman data were available. There are no obvious deviations from ideality in the compression of the intermolecular contacts.

High-pressure phase transitions are far from uncommon in molecular materials, but unless there is a change in space group or a discontinuity in the unit cell dimensions or other structural features, it can be difficult to discern a clear structural difference between the two phases. Both polymorphs of **1** show subtle, second order, isosymmetric phase transitions. The results obtained here demonstrate the great sensitivity of Raman spectroscopy to such transitions and suggest that the trends in the occupied and unoccupied volumes of the crystal structure together with deviations from ideal behavior in the intermolecular contacts can both help identify their structural signatures. These results mirror similar results obtained for the aromatic hydrocarbon naphthalene, where trends in occupied volume with pressure revealed^38^ the structural effects of a phase transition which had been debated in the literature since Bridgman’s first investigation in 1938,^42^ reconciling crystallographic and spectroscopic data. The present study also demonstrates the limitations of this approach if a transition occurs at relatively low pressure before a clear volume trend or equation of state can be established.

## Supporting Information Available

A collection of the supplementary figures and tables referred to in the text (PDF)
Movies in GIF format showing the compression of both phases viewed along the three crystallographic axes and compression of the voids (ZIP)
