# Supplementary material for: The Effect of High Pressure on Polymorphs of a Derivative of Blatter’s Radical: Identification of the Structural Signatures of Subtle Phase Transitions
Source: Cryst Growth Des. 2023 Jan 30;23(3):1915–24. doi: 10.1021/acs.cgd.2c01422 (PMC9983015; doi:10.1021/acs.cgd.2c01422)
Supplement: Supplementary file 1 — cg2c01422_si_001.pdf [file cg2c01422_si_001.pdf]

SUPPLEMENTARY INFORMATION

The Effect of High Pressure on Polymorphs of a Derivative of Blatter's Radical: Identification of the Structural Signatures of Subtle Phase Transitions

Edward T. Broadhurst,[a†] Cameron J. G. Wilson,[a] Georgia A. Zissimou,[b] Mayra Alexandra Padrón Gómez,[c] Daniel Militão Vasconcelos,[c] Christos P. Constantinides,[d] Panayiotis A. Koutentis,[b] Alejandro P. Ayala[c*] and Simon Parsons[a*]

[a] EaStCHEM School of Chemistry and Centre for Science at Extreme Conditions, The University of Edinburgh, King's Buildings, West Mains Road, Edinburgh, EH9 3FJ, Scotland.

[b] Department of Chemistry, University of Cyprus, 20537, 1678 Nicosia, Cyprus.

c Federal University of Ceará, Physics Department, 65455-900, Fortaleza (CE), Brasil

d Department of Natural Sciences, University of Michigan-Dearborn, 4901 Evergreen Road, Dearborn, Michigan, 48128-1491, USA.

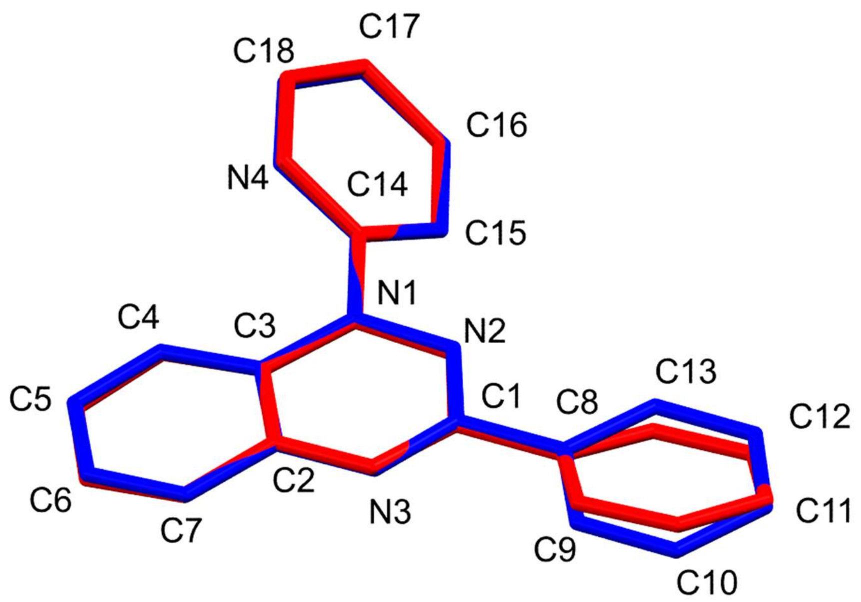

Figure S1. Overlay of molecules in **1α** (red) and **1β** (blue).

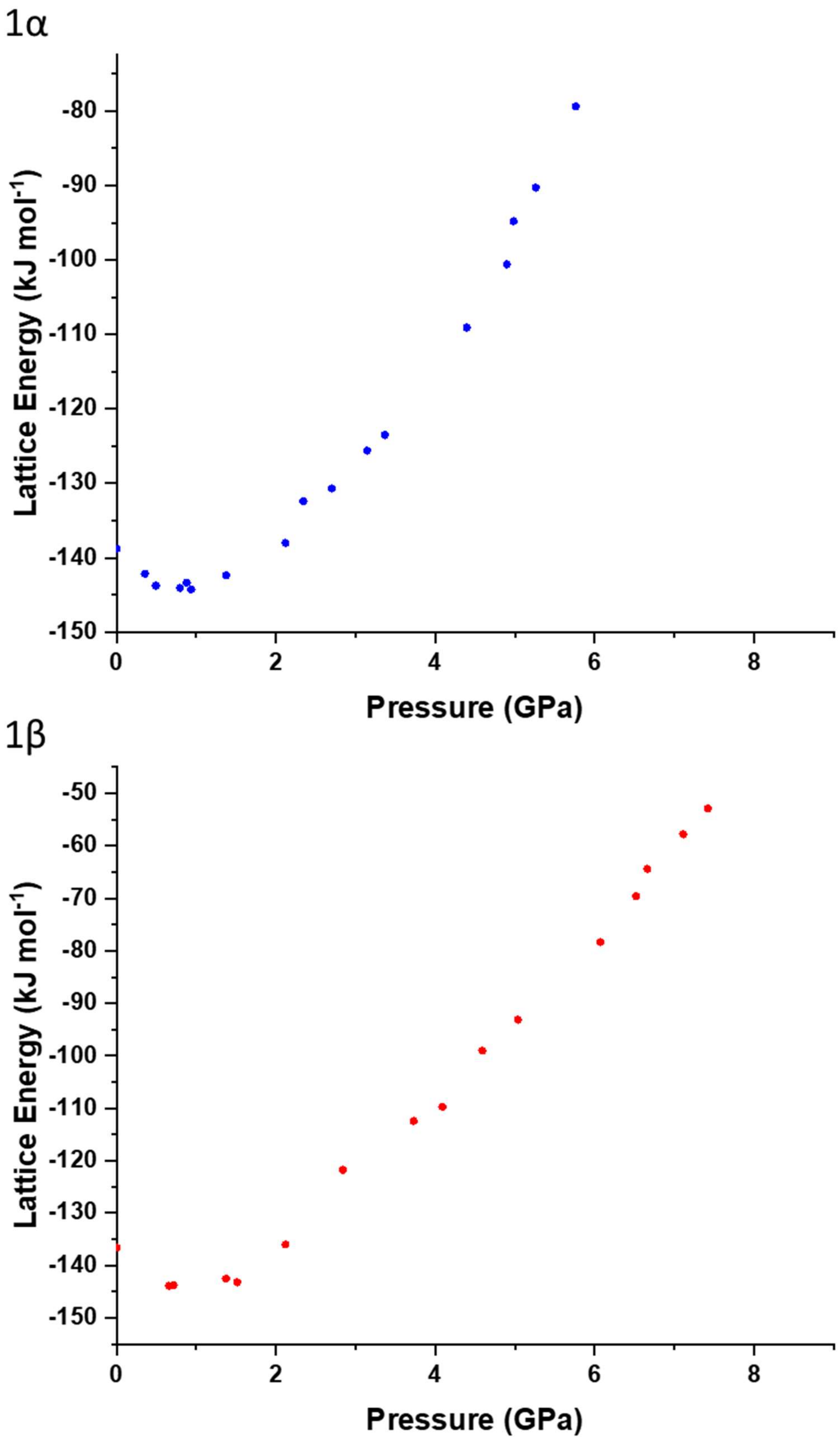

Figure S2. Lattice energies calculated using the PIXEL method with increasing pressure. Blue = **1α**, red = **1β**.

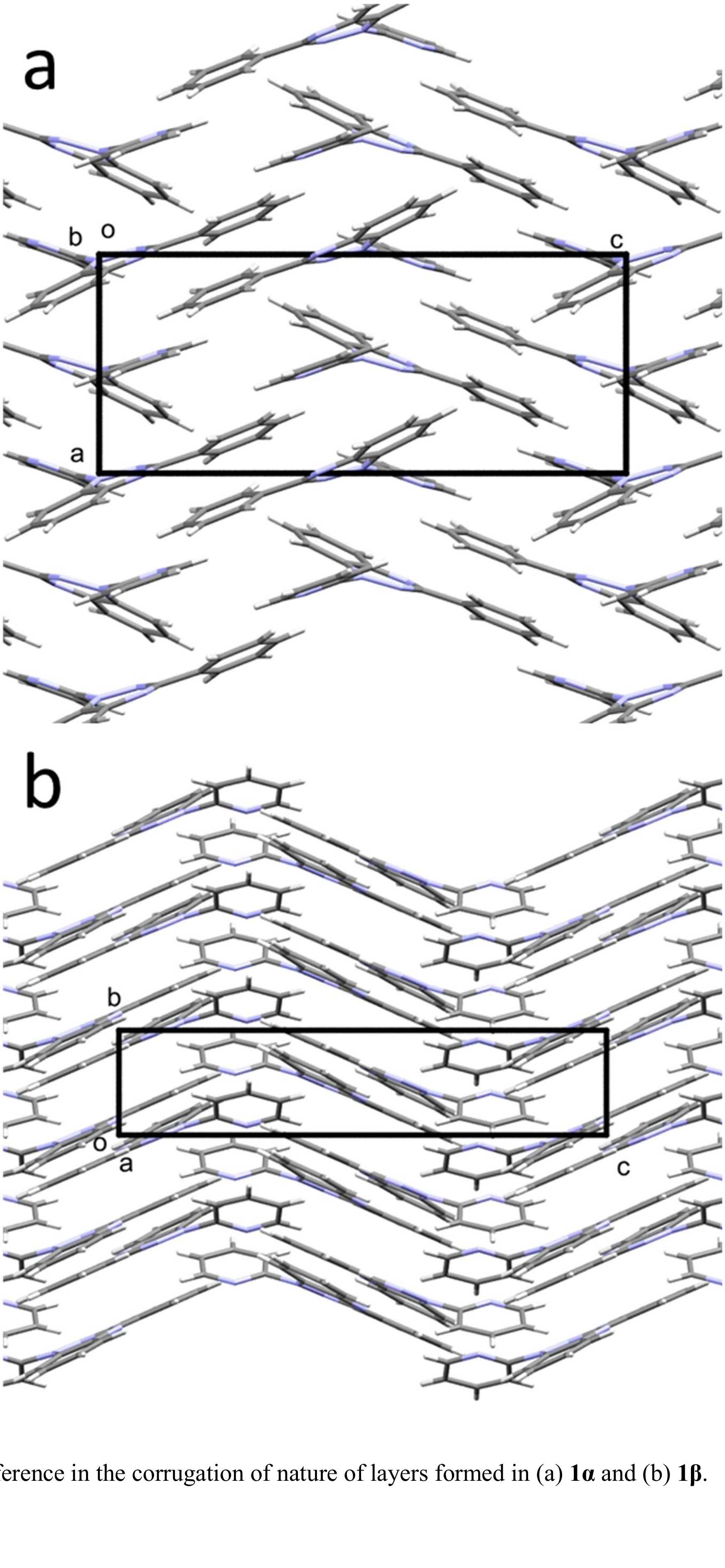

Figure S3. Difference in the corrugation of nature of layers formed in (a) **1α** and (b) **1β**.

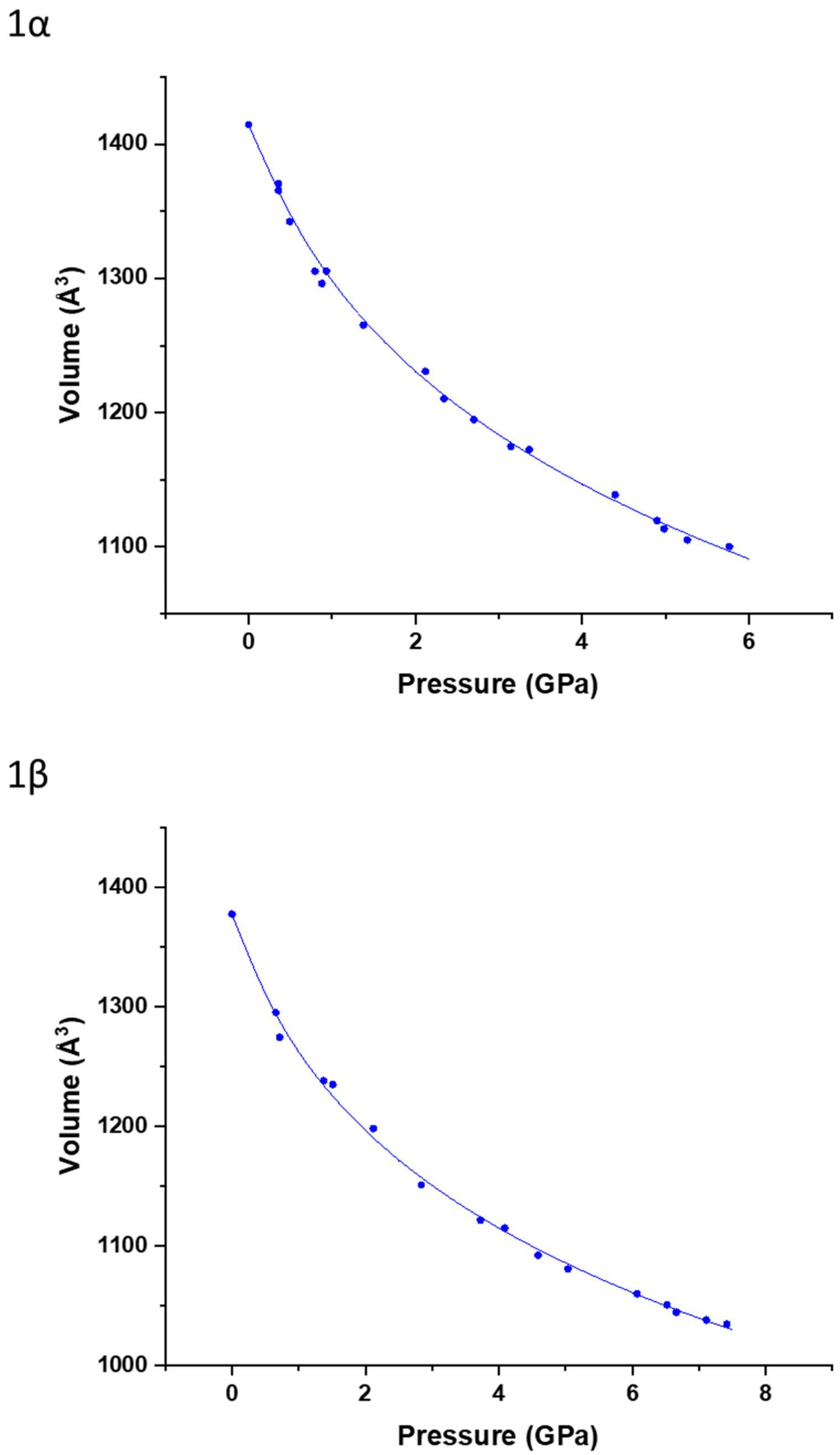

Figure S4. Variation of unit-cell volume with pressure for **1α** and **1β**. The trend lines are third order Birch −Murnaghan equations of state. Error bars lie within the symbols.

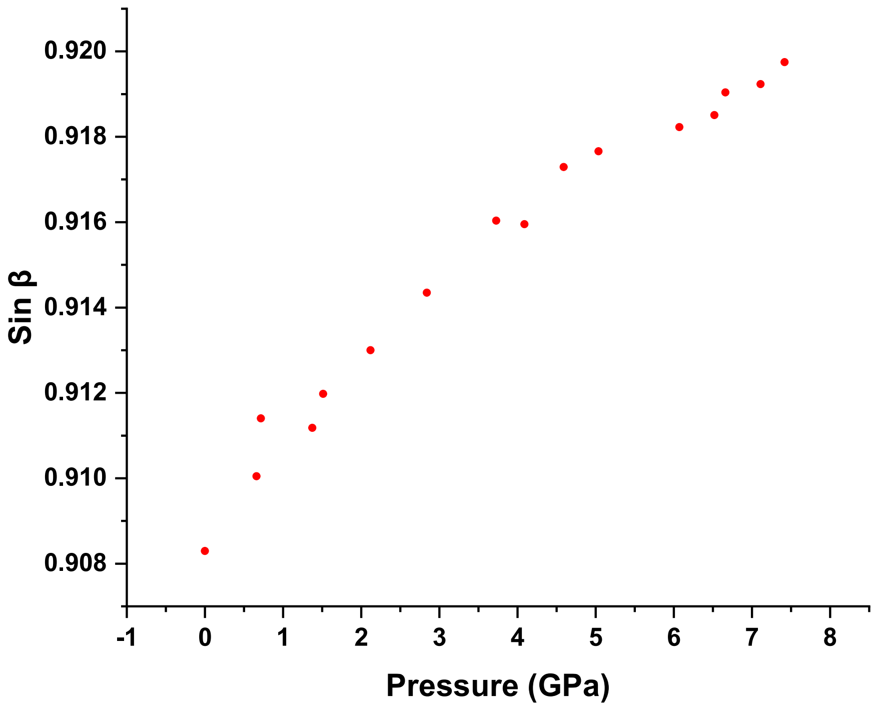

Figure S5. A plot of sin β with increasing pressure for **1β**.

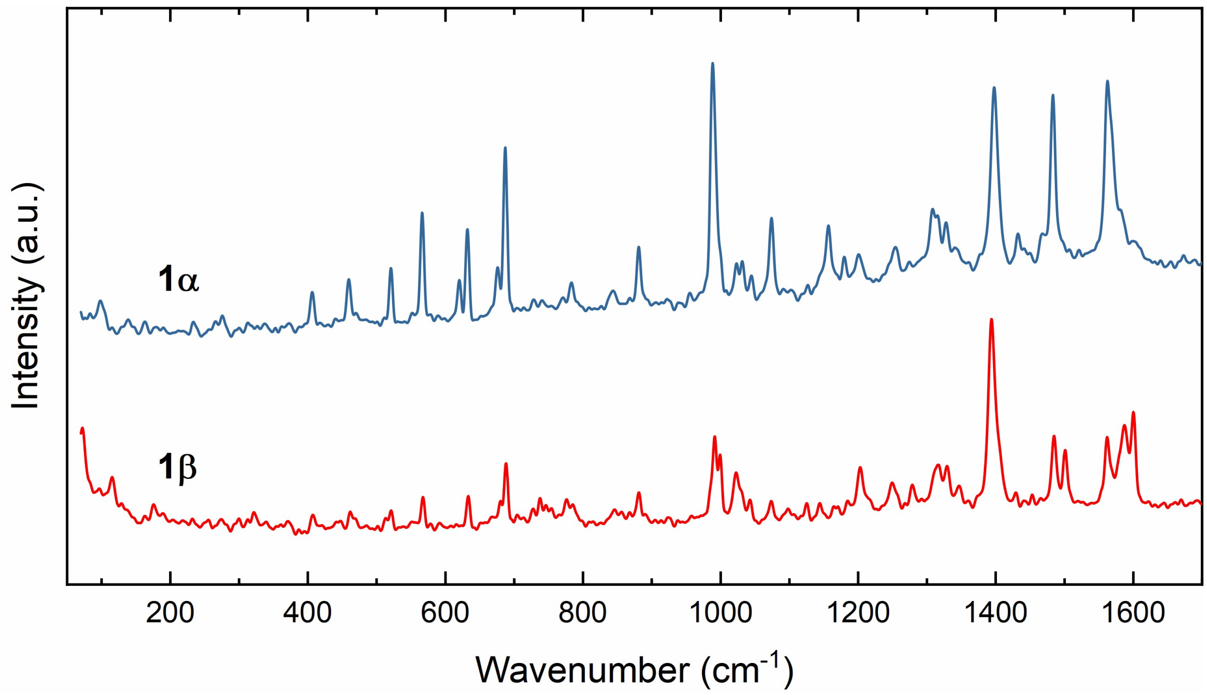

Figure S6. Raman spectra of **1α** and **1β** at ambient pressure.

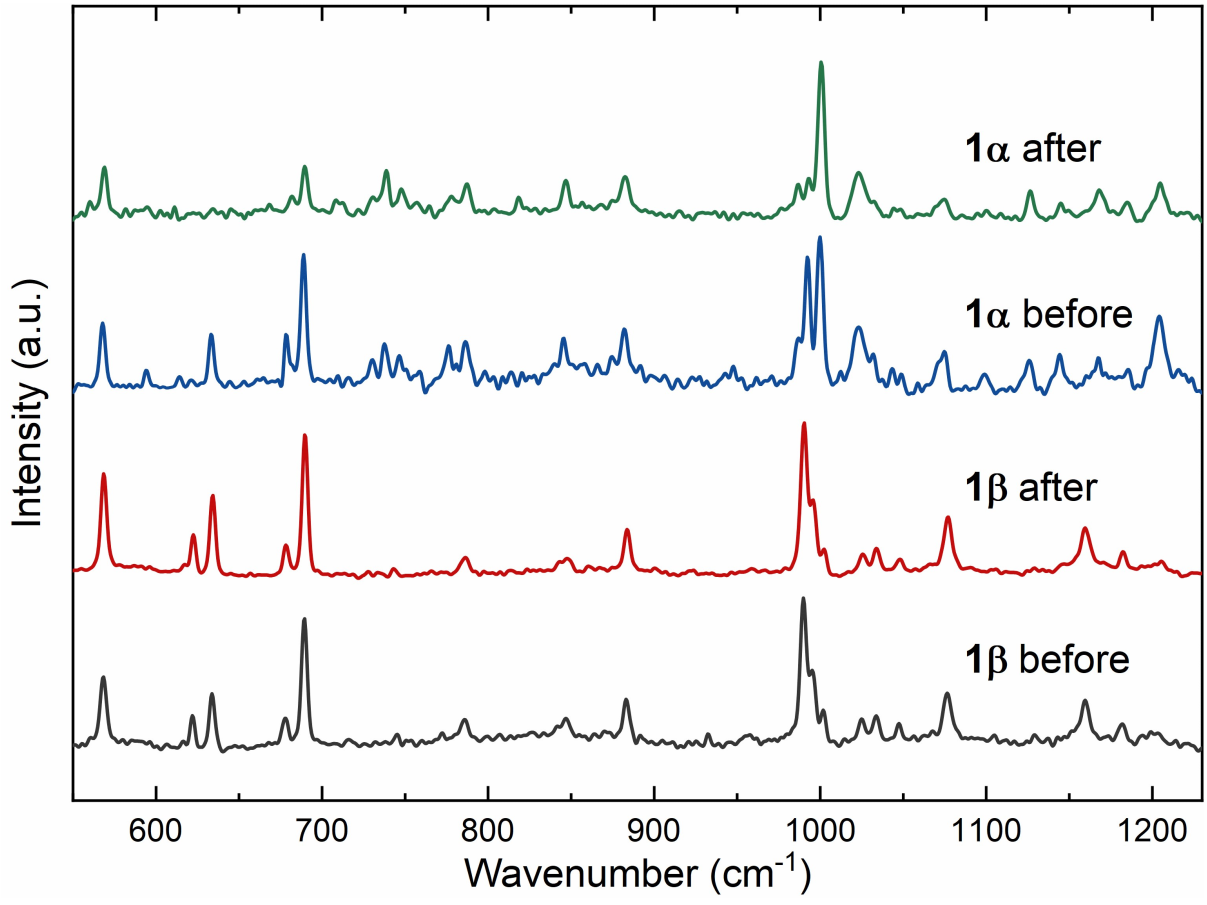

Figure S7. Comparison of the Raman spectra of **1α** and **1β** at ambient conditions before application of pressure and after pressure release following amorphization at 5.36 and 5.43 GPa, respectively.

1α

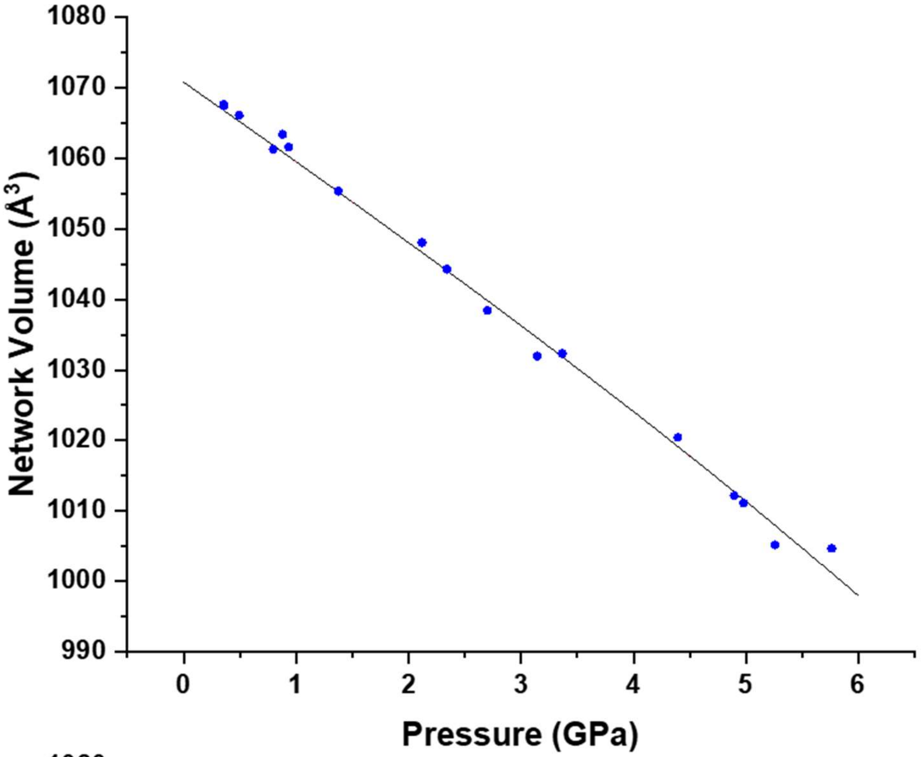

1β

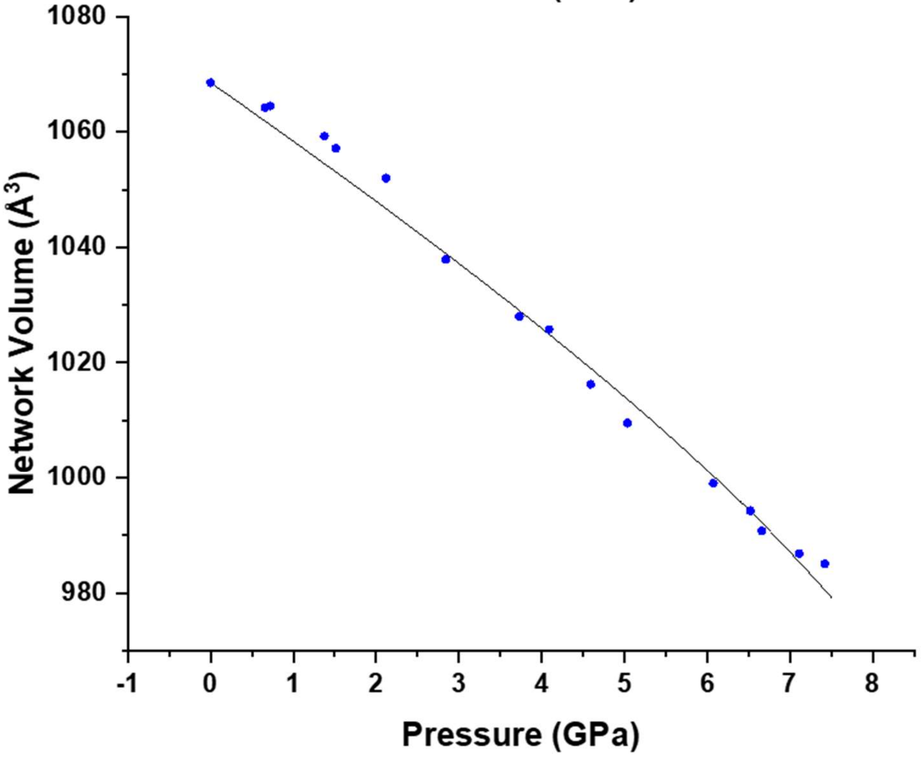

Figure S8. Third order Birch −Murnaghan equation of state fit to network volumes for each polymorph.

1α

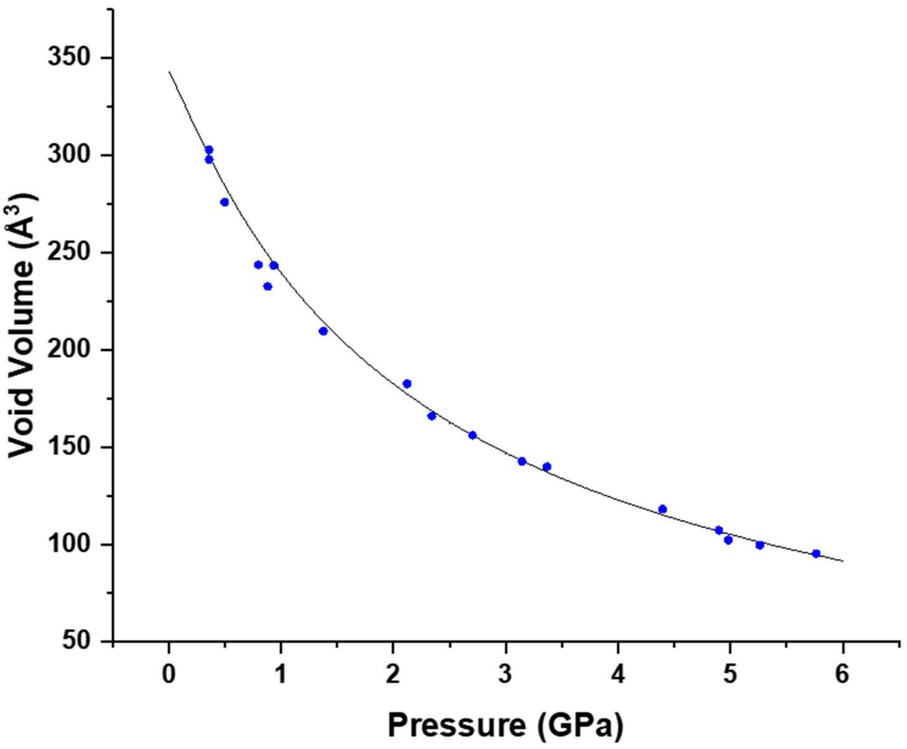

1β

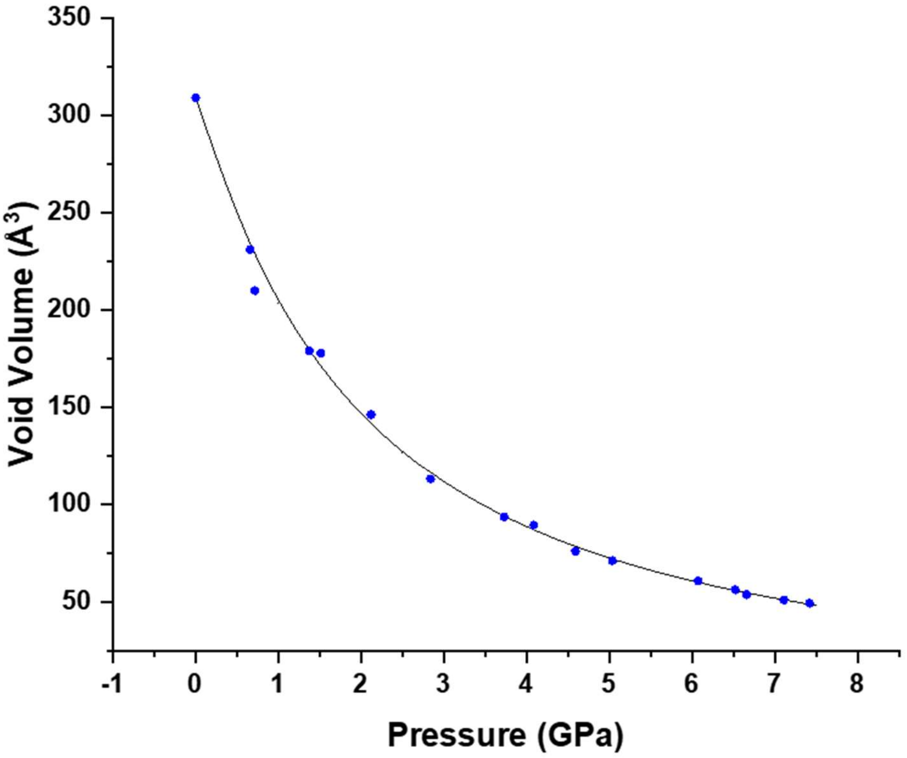

Figure S9. Third order Vinet equation of state fit to void volumes for each polymorph.

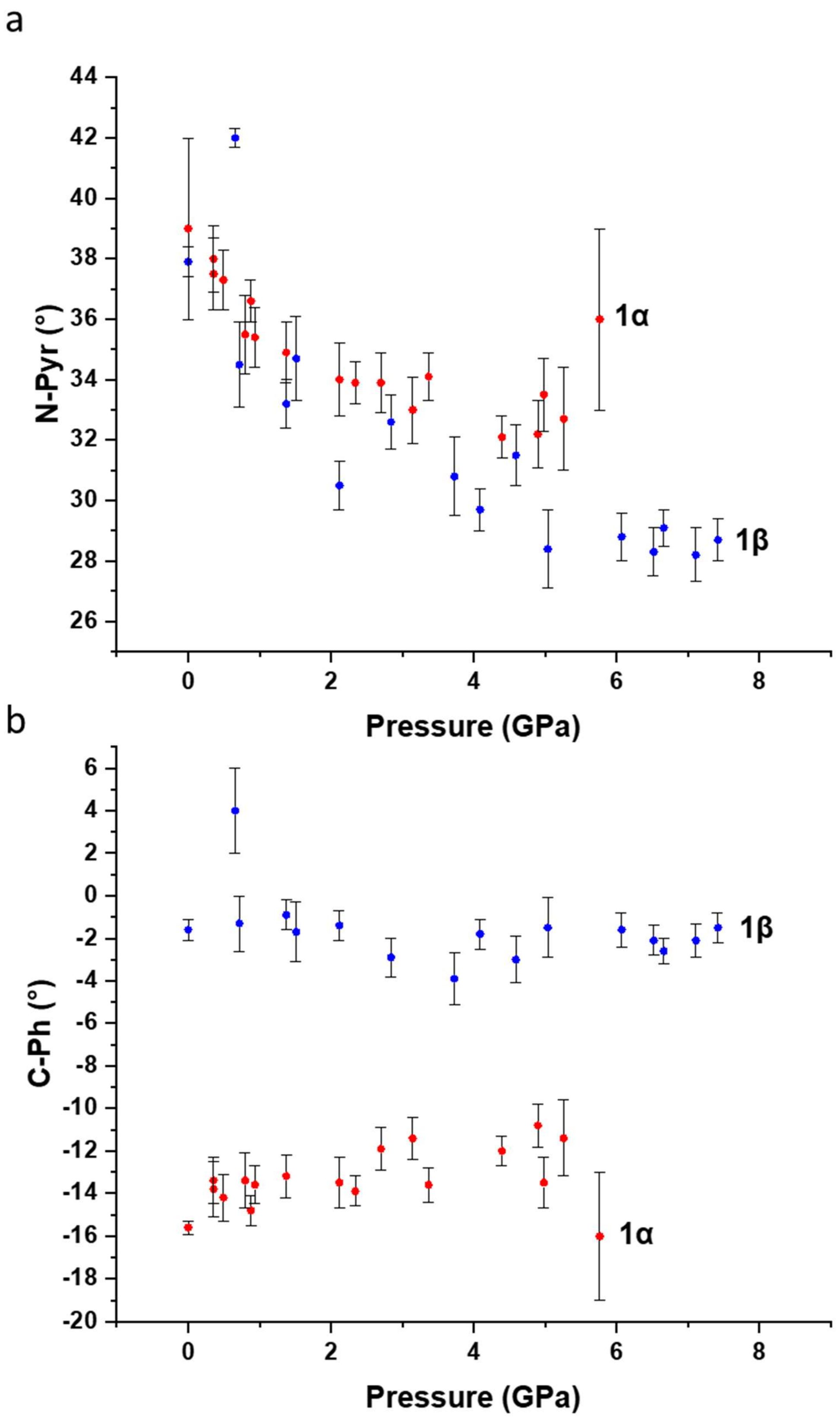

Figure S10. Torsional angles of the ring substituents with pressure on each polymorph. Calculated using PLATON. a) C3−N1−C14−N4 (the N-Pyr torsion) and b) C3−N1−C8−C9 (the C-Ph torsion).

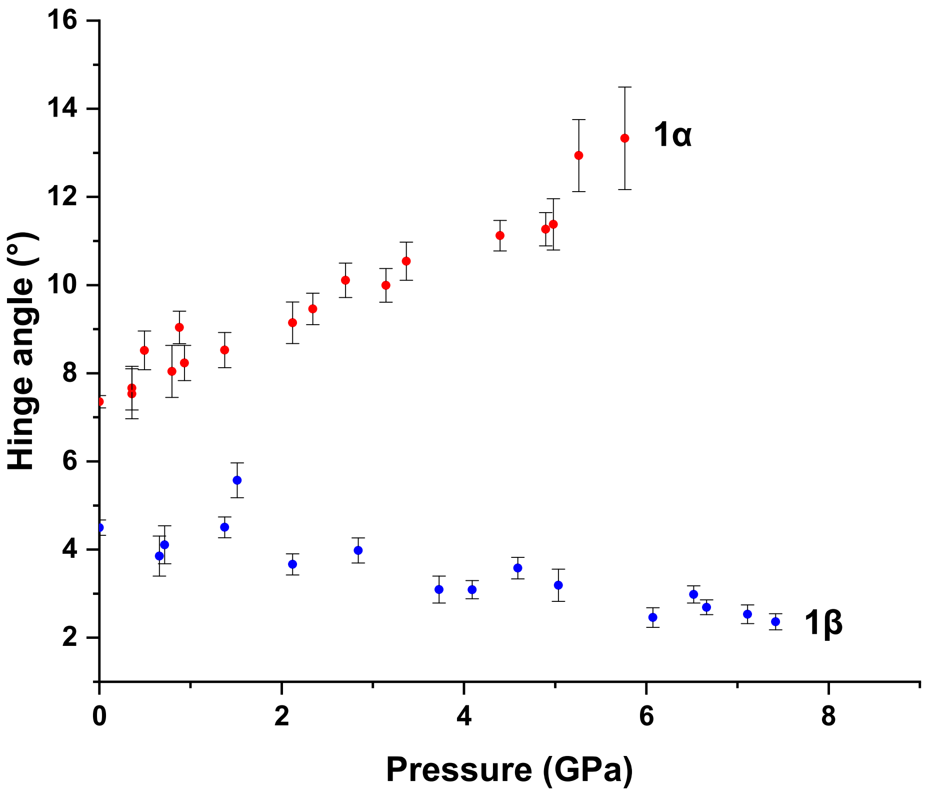

Figure S11. The "hinge" angle on the triazinyl core (N1−N2−C1−N3∠N1−C3−C2−N3) with increasing pressure for **1α** and **1β**.

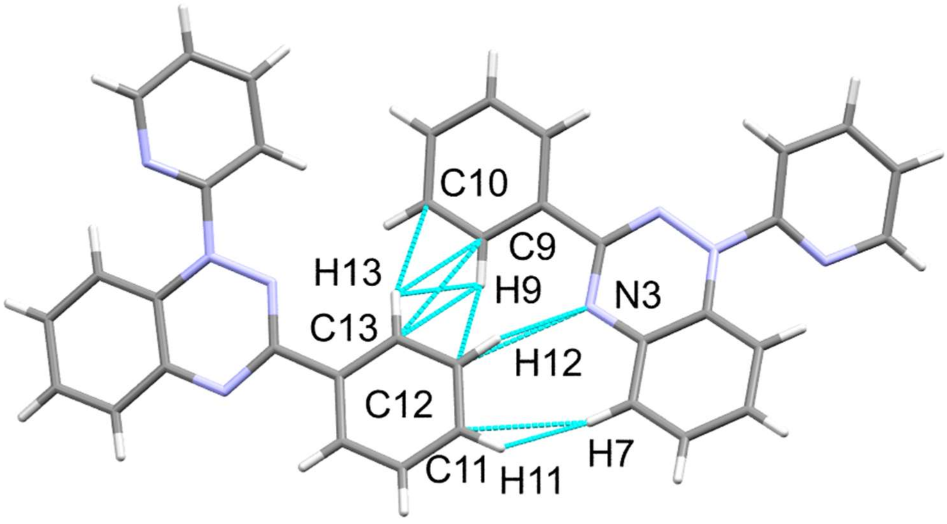

Figure S12. Short contacts in interaction B in **1α** at 5.76 GPa.

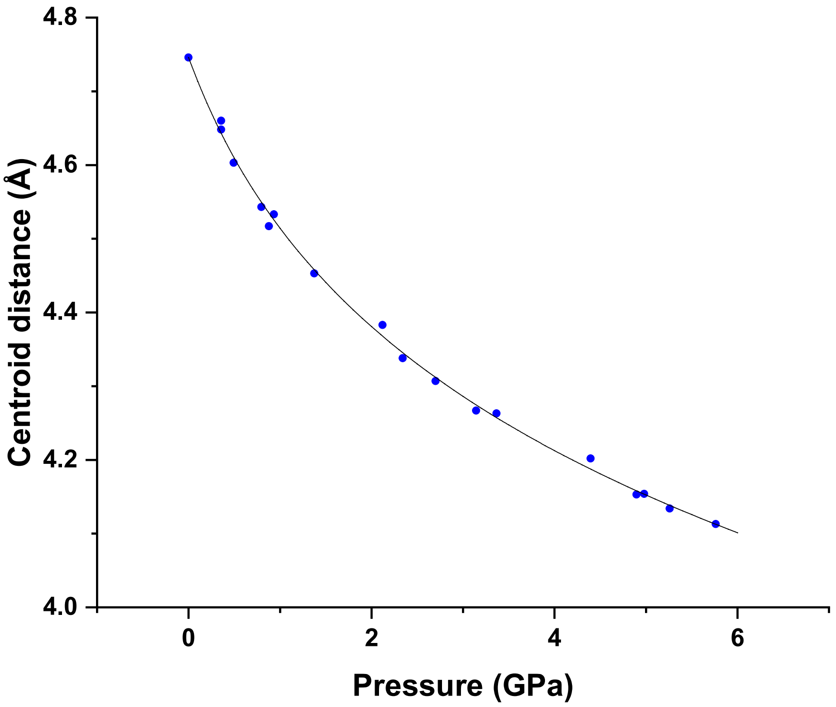

Figure S13. Linear Modulus (M_0) fit (black line) to centroid distance (blue dots) with pressure for contact A in **1α**.

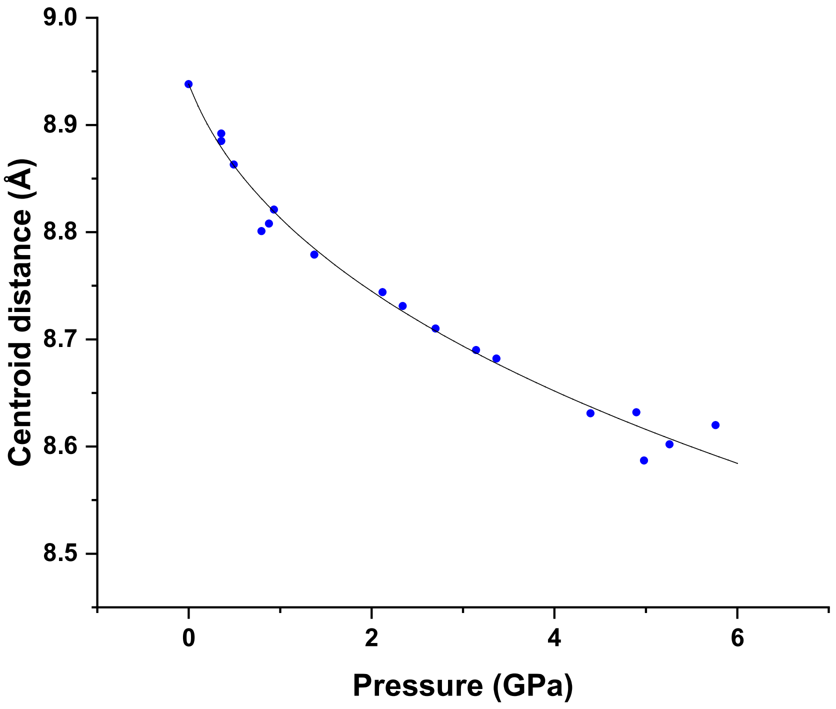

Figure S14. Linear Modulus (M_0) fit (black line) to centroid distance (blue dots) with pressure for contact B in **1α**.

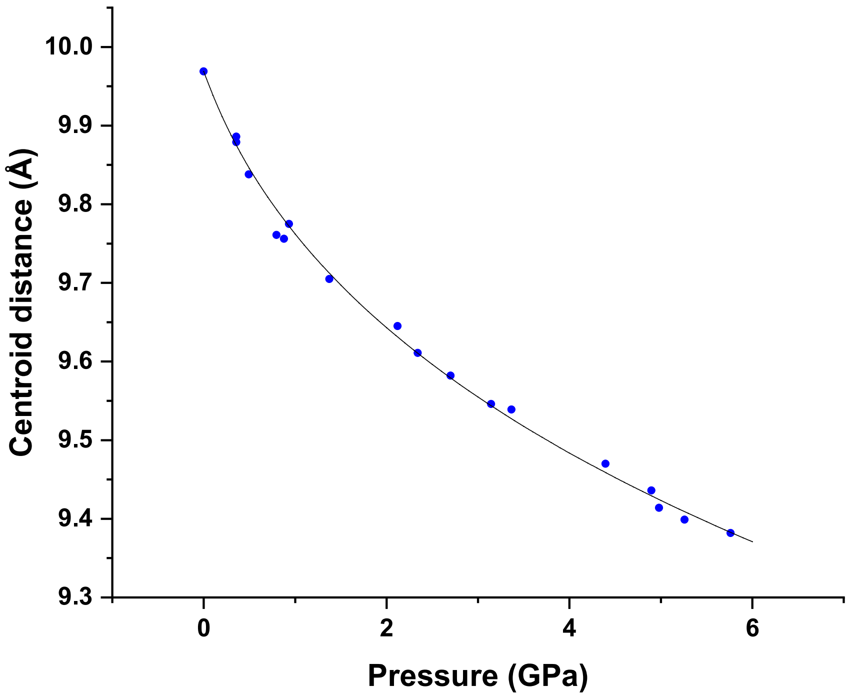

Figure S15. Linear Modulus (M_0) fit (black line) to centroid distance (blue dots) with pressure for contact C in **1α**.

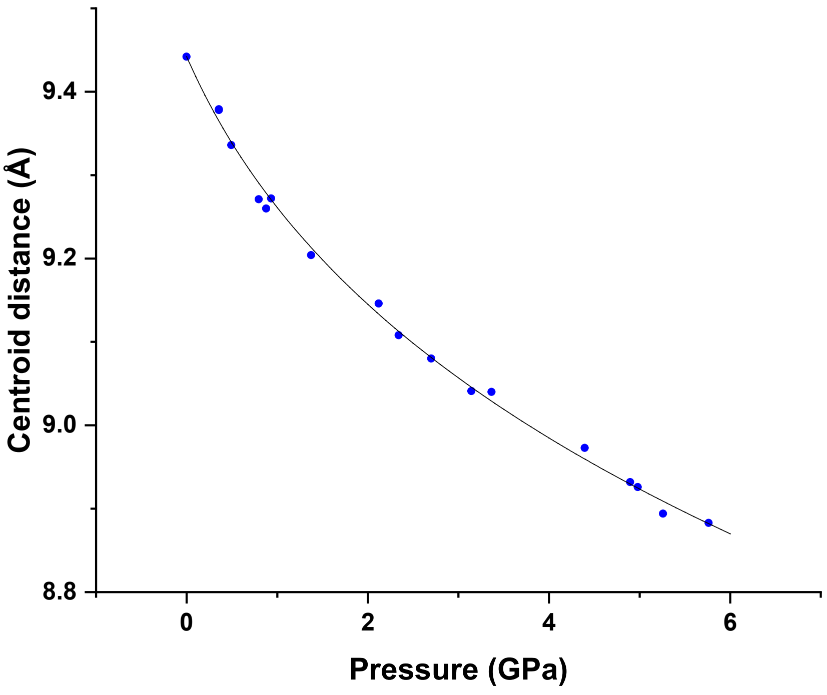

Figure S16. Linear Modulus (M_0) fit (black line) to centroid distance (blue dots) with pressure for contact D in **1α**.

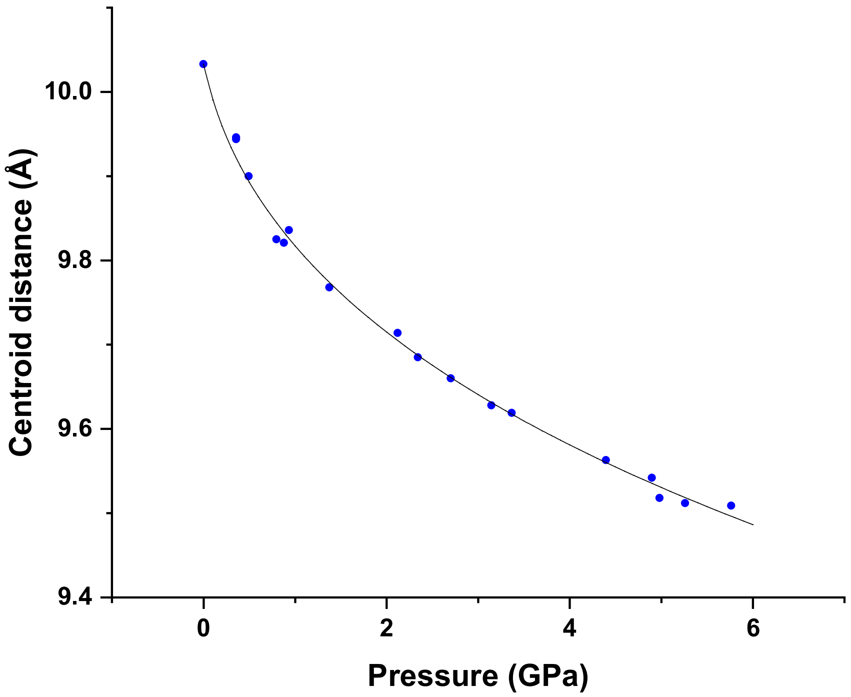

Figure S17. Linear Modulus (M_0) fit (black line) to centroid distance (blue dots) with pressure for contact E in **1α**.

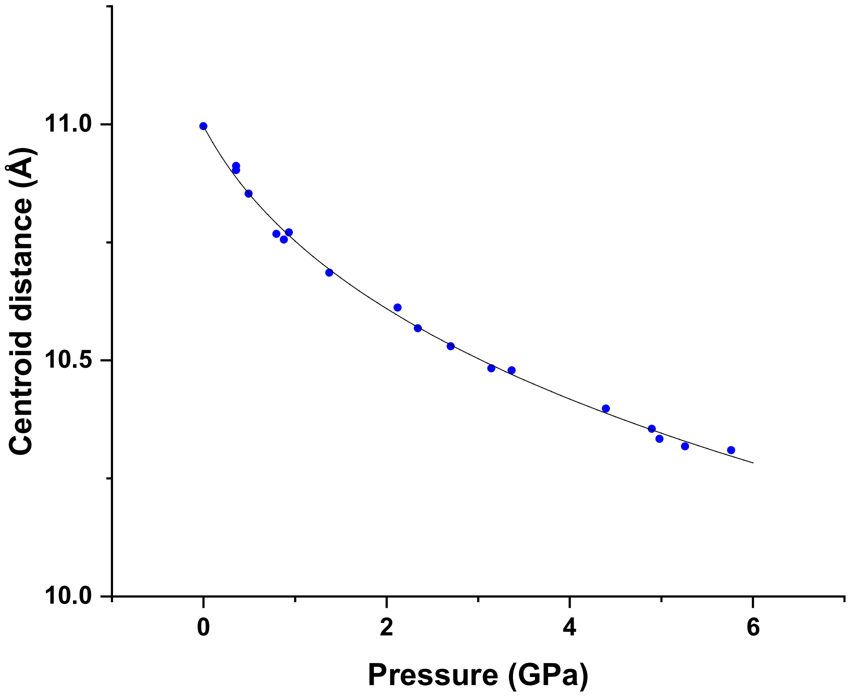

Figure S18. Linear Modulus (M_0) fit (black line) to centroid distance (blue dots) with pressure for contact F in **1α**.

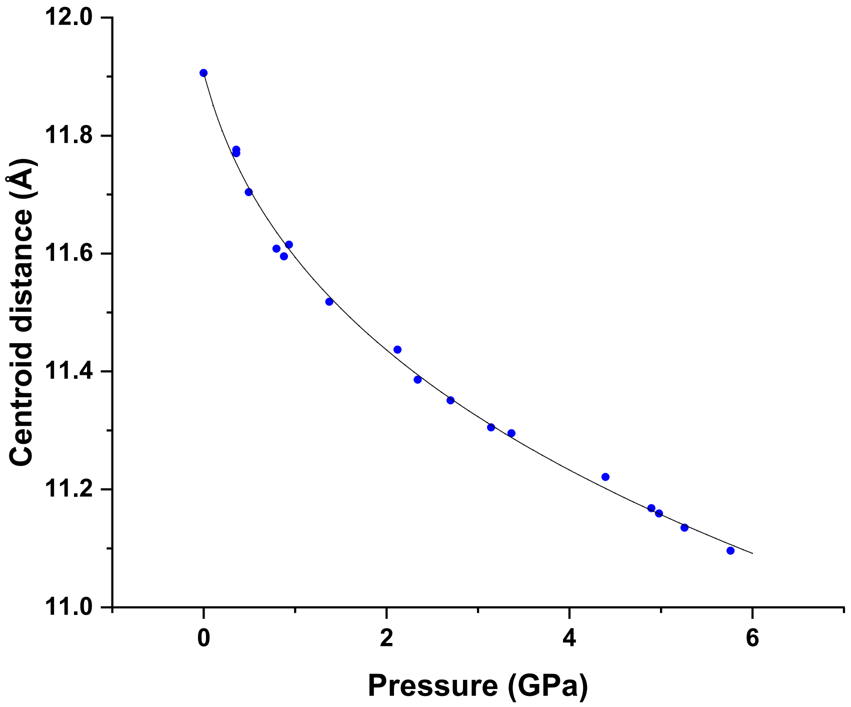

Figure S19. Linear Modulus (M_0) fit (black line) to centroid distance (blue dots) with pressure for contact G in **1α**.

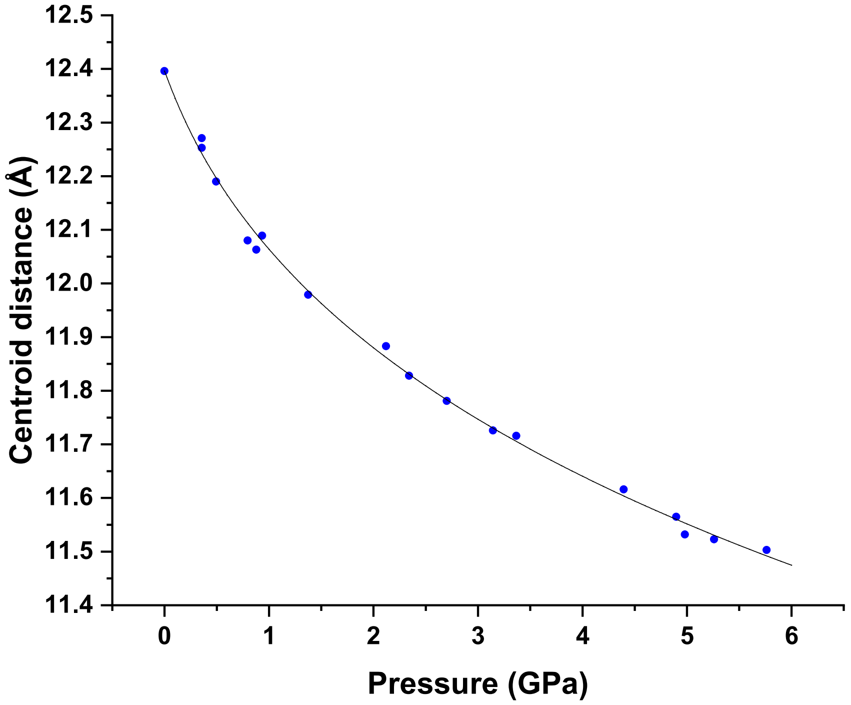

Figure S20. Linear Modulus (M_0) fit (black line) to centroid distance (blue dots) with pressure for contact H in **1α**.

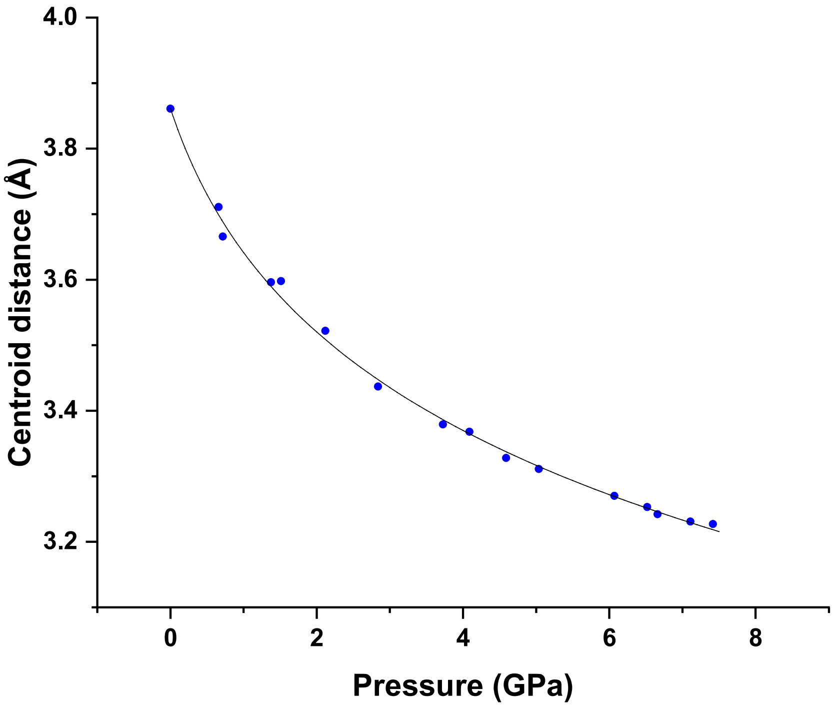

Figure S21. Linear Modulus (M_0) fit (black line) to centroid distance (blue dots) with pressure below 2.12 GPa for contact A in **1β**.

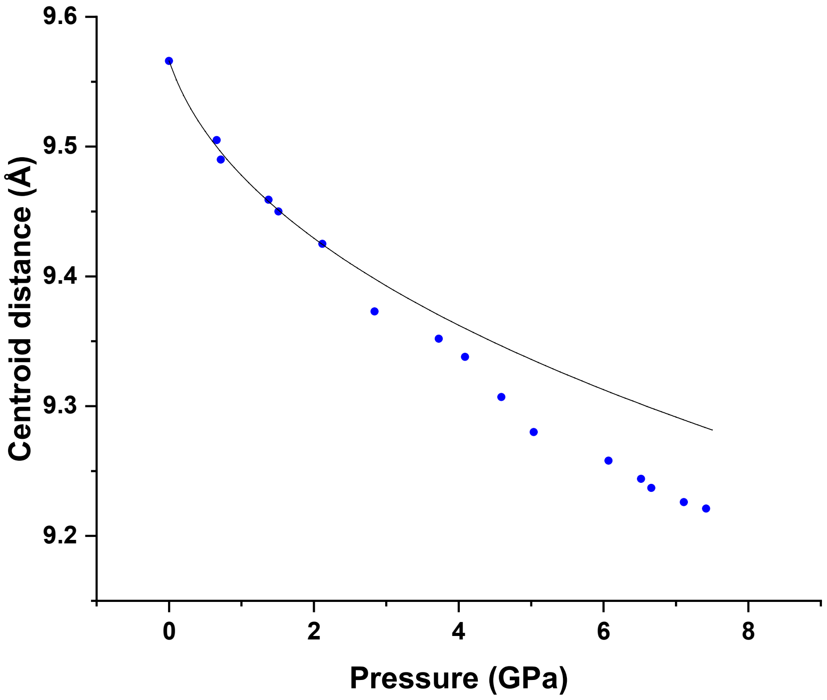

Figure S22. Linear Modulus (M_0) fit (black line) to centroid distance (blue dots) with pressure below 2.12 GPa for contact B in **1β**.

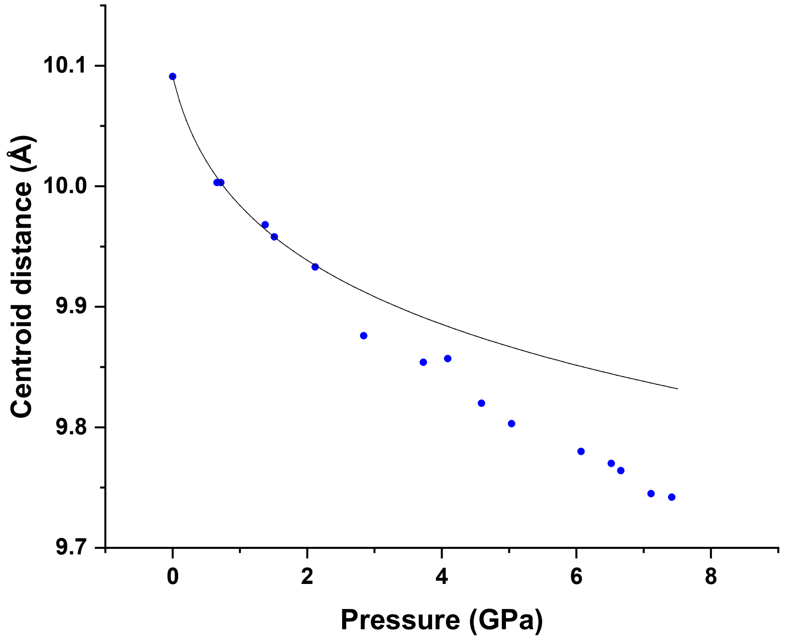

Figure S23. Linear Modulus (M_0) fit (black line) to centroid distance (blue dots) with pressure below 2.12 GPa for contact C in **1β**.

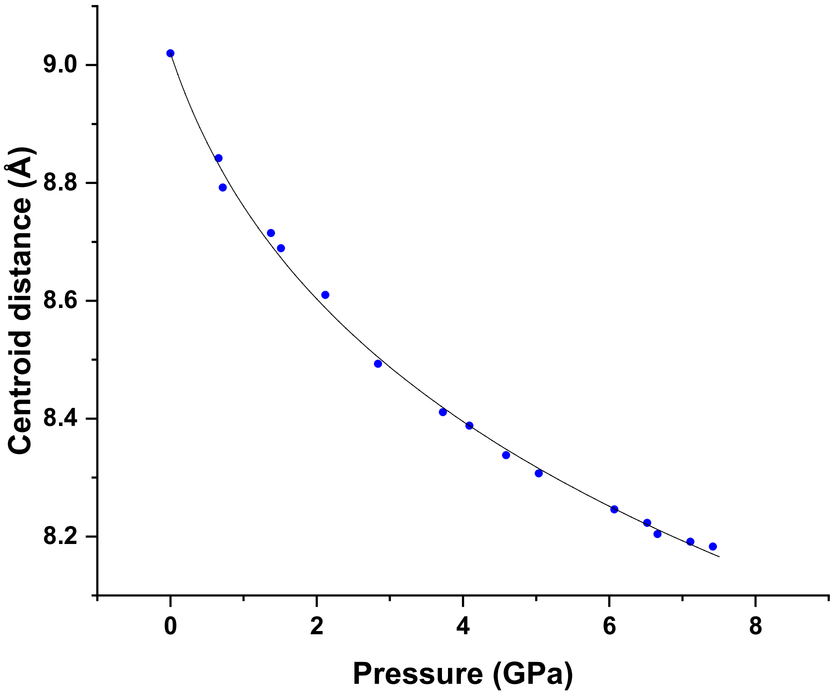

Figure S24. Linear Modulus (M_0) fit (black line) to centroid distance (blue dots) with pressure below 2.12 GPa for contact D in **1β**.

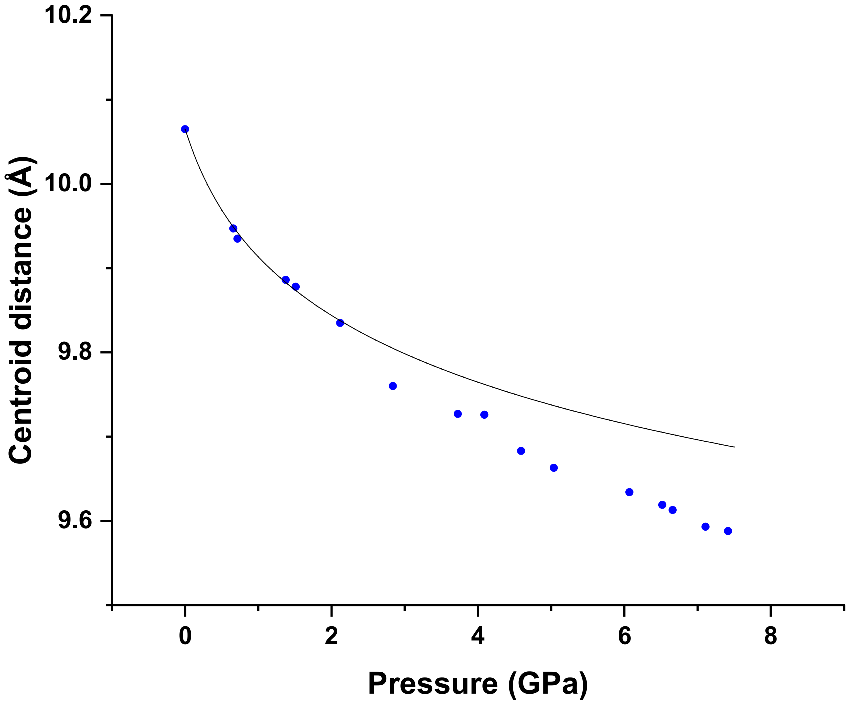

Figure S25. Linear Modulus (M_0) fit (black line) to centroid distance (blue dots) with pressure below 2.12 GPa for contact E in **1β**.

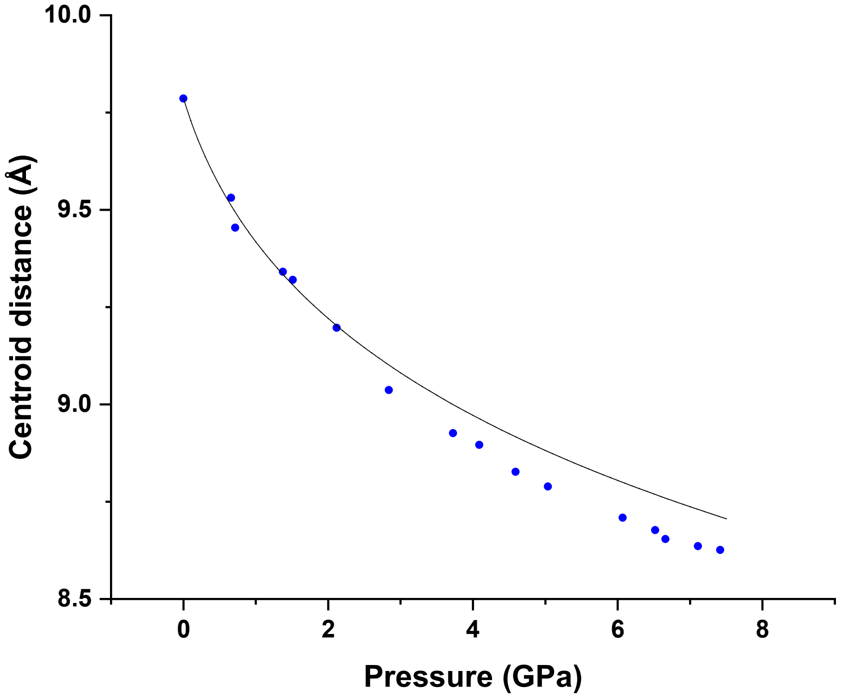

Figure S26. Linear Modulus (M_0) fit (black line) to centroid distance (blue dots) with pressure below 2.12 GPa for contact F in **1β**.

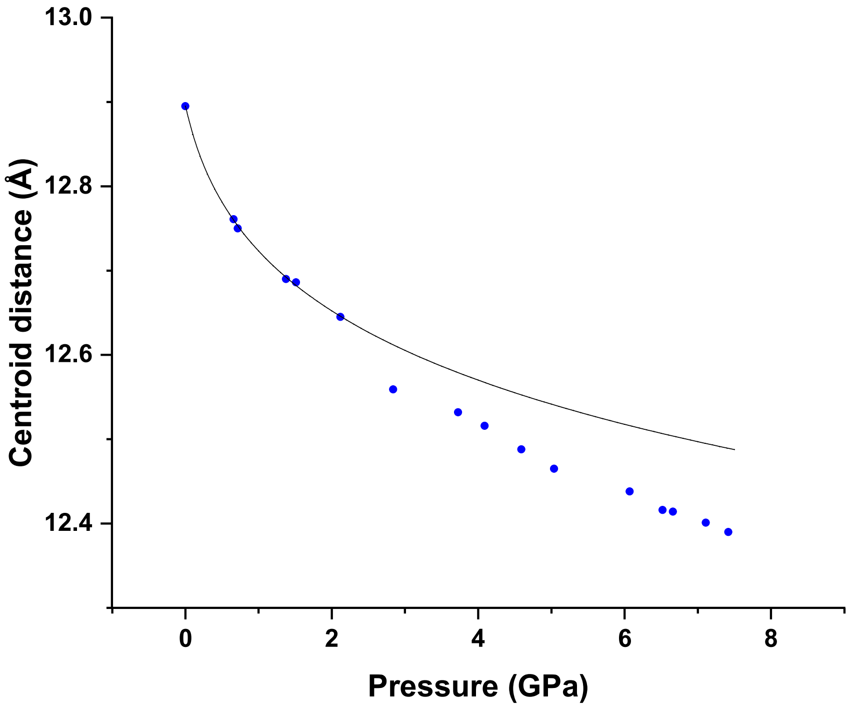

Figure S27. Linear Modulus (M_0) fit (black line) to centroid distance (blue dots) with pressure below 2.12 GPa for contact G in **1β**.

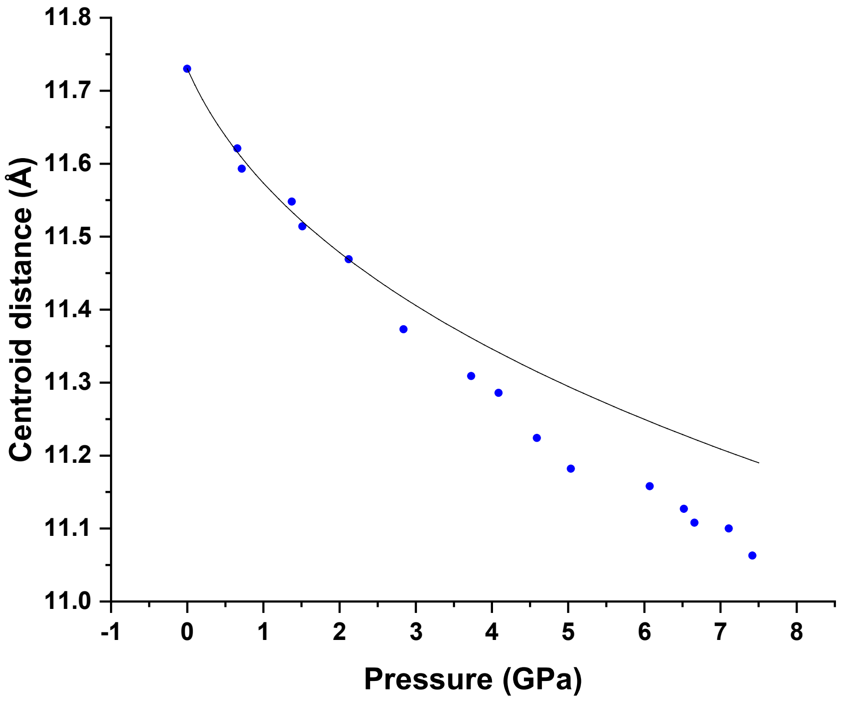

Figure S28. Linear Modulus (M_0) fit (black line) to centroid distance (blue dots) with pressure below 2.12 GPa for contact H in **1β**.

Table S1. Experimental details of the high−pressure collection of polymorph **1α**. For all structures: $C_{18}H_{13}N_4$, M_r = 285.32, orthorhombic, $P2_12_12_1$, Z = 4. Experiments were carried out at 298 K with Mo $K\alpha$ radiation using a Bruker SMART APEX2 area detector. H-atom parameters were constrained.

**Pressure (GPa)**	**0.00**	**0.36**	**0.36**	**0.49**
Crystal #	1	2	1	2
a, b, c (Å)	7.3061 (2), 10.9963 (3), 17.6057 (6)	7.1946 (13), 10.9123 (14), 17.456 (2)	7.180 (2), 10.903 (2), 17.446 (8)	7.1173 (9), 10.853 (3), 17.377 (3)
V (Å^3)	1414.44 (7)	1370.4 (3)	1365.6 (8)	1342.3 (4)
μ (mm^{-1})	0.08	0.09	0.09	0.09
Crystal size (mm)	0.6 × 0.4 × 0.3	0.17 × 0.1 × 0.09	0.14 × 0.1 × 0.09	0.17 × 0.1 × 0.09
Absorption correction ($wR2_{bef}$, $wR2_{aft}$, max : min transmission)	0.0735, 0.0497, 0.8873	0.1708, 0.0676, 0.7191	0.1622, 0.0891, 0.6582	0.1118, 0.0533, 0.9047
T_{min}, T_{max}	0.662, 0.746	0.536, 0.745	0.487, 0.745	0.674, 0.745
No. of measured, independent and observed [$I > 2\sigma(I)$] reflections	15326, 4269, 2946	6839, 1757, 954	8006, 1458, 682	8010, 1436, 799
R_{int}	0.043	0.115	0.234	0.098
$(\sin \theta/\lambda)_{max}$ (Å^{-1})	0.713	0.623	0.595	0.624
$R[F^2 > 2\sigma(F^2)]$, $wR(F^2)$, S	0.045, 0.114, 1.06	0.067, 0.170, 0.98	0.059, 0.136, 0.96	0.059, 0.142, 0.99
Data completeness	0.99	0.63	0.60	0.53
No. of reflections	4269	1757	1458	1436
No. of parameters	200	199	199	199
No. of restraints	0	230	230	53
$\Delta\rho_{max}$, $\Delta\rho_{min}$ (e Å^{-3})	0.18, −0.14	0.22, −0.31	0.21, −0.19	0.19, −0.19

Table S1 continued

**Pressure (GPa)**	**0.80**	**0.88**	**0.93**	**1.37**
Crystal #	1	1	2	2
a, *b*, *c* (Å)	7.0285 (16), 10.7684 (17), 17.245 (6)	6.9872 (6), 10.7557 (9), 17.248 (3)	7.0147 (9), 10.7712 (7), 17.2756 (10)	6.8964 (8), 10.6859 (6), 17.1684 (9)
V (Å^3)	1305.2 (6)	1296.2 (3)	1305.3 (2)	1265.21 (18)
μ (mm^{-1})	0.09	0.09	0.09	0.09
Crystal size (mm)	0.14 × 0.1 × 0.09	0.14 × 0.1 × 0.09	0.17 × 0.1 × 0.09	0.17 × 0.1 × 0.09
Absorption correction ($wR2_{bef}$, $wR2_{aft}$, max : min transmission)	0.1168, 0.0661, 0.8665	0.0786, 0.0493, 0.8992	0.0783, 0.0537, 0.8791	0.0824, 0.0491, 0.8970
T_{min}, T_{max}	0.646, 0.745	0.670, 0.745	0.655, 0.745	0.669, 0.745
No. of measured, independent and observed [$I > 2\sigma(I)$] reflections	6758, 1474, 684	6698, 986, 775	7820, 1355, 1031	6670, 1278, 1030
R_{int}	0.162	0.048	0.053	0.041
$(\sin \theta/\lambda)_{max}$ (Å^{-1})	0.626	0.625	0.628	0.625
$R[F^2 > 2\sigma(F^2)]$, $wR(F^2)$, S	0.066, 0.165, 0.96	0.033, 0.067, 1.04	0.043, 0.105, 1.08	0.041, 0.102, 1.06
Data completeness	0.55	0.34	0.50	0.49
No. of reflections	1474	986	1355	1278
No. of parameters	199	199	199	199
No. of restraints	230	230	230	230
$\Delta\rho_{max}$, $\Delta\rho_{min}$ (e Å^{-3})	0.23, −0.22	0.11, −0.15	0.12, −0.13	0.12, −0.14

Table S1 continued

**Pressure (GPa)**	**2.12**	**2.34**	**2.70**	**3.14**
Crystal #	2	1	2	2
a, b, c (Å)	6.7901 (10), 10.6124 (7), 17.0808 (11)	6.7240 (6), 10.5677 (8), 17.036 (3)	6.6777 (7), 10.5296 (5), 16.9936 (9)	6.6134 (8), 10.4833 (6), 16.9451 (9)
V (Å^3)	1230.8 (2)	1210.5 (3)	1194.88 (15)	1174.81 (17)
μ (mm^{-1})	0.10	0.10	0.10	0.10
Crystal size (mm)	0.17 × 0.1 × 0.09	0.14 × 0.1 × 0.09	0.17 × 0.1 × 0.09	0.17 × 0.1 × 0.09
Absorption correction ($wR2_{bef}$, $wR2_{aft}$, max : min transmission)	0.1672, 0.0953, 0.8107	0.0783, 0.0493, 0.8952	0.0718, 0.0497, 0.9049	0.0716, 0.0489, 0.8900
T_{min}, T_{max}	0.604, 0.745	0.667, 0.745	0.675, 0.745	0.663, 0.745
No. of measured, independent and observed [$I > 2\sigma(I)$] reflections	6631, 1255, 924	6019, 967, 756	6295, 1212, 989	5845, 1172, 938
R_{int}	0.061	0.050	0.041	0.043
θ_{max} (°)	26.4	26.3	26.3	26.4
$(\sin \theta/\lambda)_{\mathrm{max}}$ (Å^{-1})	0.624	0.624	0.624	0.625
$R[F^2 > 2\sigma(F^2)]$, $wR(F^2)$, S	0.047, 0.122, 1.05	0.030, 0.061, 1.01	0.041, 0.099, 1.07	0.043, 0.095, 1.08
Data completeness	0.50	0.39	0.50	0.49
No. of reflections	1255	967	1212	1172
No. of parameters	199	199	199	199
No. of restraints	230	230	53	89
$\Delta\rho_{\mathrm{max}}$, $\Delta\rho_{\mathrm{min}}$ (e Å^{-3})	0.17, −0.17	0.11, −0.11	0.15, −0.15	0.15, −0.19

Table S1 continued

**Pressure (GPa)**	**3.37**	**4.40**	**4.90**	**4.98**
Crystal #	1	1	2	1
a, *b*, *c* (Å)	6.6104 (9), 10.4790 (12), 16.927 (4)	6.5075 (5), 10.3977 (8), 16.831 (3)	6.4369 (7), 10.3547 (5), 16.7986 (9)	6.4345 (13), 10.334 (2), 16.746 (6)
V (Å^3)	1172.5 (4)	1138.9 (2)	1119.66 (15)	1113.5 (5)
μ (mm^{-1})	0.10	0.10	0.11	0.11
Crystal size (mm)	0.14 × 0.1 × 0.09	0.14 × 0.1 × 0.09	0.17 × 0.1 × 0.09	0.14 × 0.1 × 0.09
Absorption correction ($wR2_{bef}$, $wR2_{aft}$, max : min transmission)	0.1752, 0.0565, 0.8920	0.1263, 0.0650, 0.8095	0.1278, 0.0613, 0.8787	0.1551, 0.0914, 0.7405
T_{min}, T_{max}	0.665, 0.746	0.603, 0.745	0.655, 0.745	0.552, 0.745
No. of measured, independent and observed [$I > 2\sigma(I)$] reflections	6873, 991, 717	5635, 979, 728	5555, 1099, 882	5658, 891, 576
R_{int}	0.081	0.058	0.049	0.115
$R[F^2 > 2\sigma(F^2)]$, $wR(F^2)$, S	0.038, 0.070, 1.05	0.035, 0.077, 1.04	0.039, 0.098, 1.09	0.053, 0.130, 1.06
Data completeness	0.41	0.42	0.48	0.39
No. of reflections	991	979	1099	891
No. of parameters	199	199	194	199
No. of restraints	230	230	236	230
$\Delta\rho_{\mathrm{max}}$, $\Delta\rho_{\mathrm{min}}$ (e Å^{-3})	0.15, −0.14	0.16, −0.16	0.16, −0.19	0.19, −0.18

Table S1 continued

**Pressure (GPa)**	**5.26**	**5.76**
Crystal #	1	2
a, b, c (Å)	6.395 (2), 10.318 (4), 16.748 (10)	6.3781 (13), 10.3097 (12), 16.731 (2)
V (Å^3)	1105.1 (9)	1100.2 (3)
μ (mm^{-1})	0.11	0.11
Crystal size (mm)	0.14 × 0.1 × 0.09	0.17 × 0.1 × 0.09
Absorption correction ($wR2_{bef}$, $wR2_{aft}$, max : min transmission)	0.1153, 0.0915, 0.7690	0.0810, 0.0566, 0.8260
T_{min}, T_{max}	0.573, 0.745	0.616, 0.745
No. of measured, independent and observed [$I > 2\sigma(I)$] reflections	5279, 545, 451	2215, 688, 485
R_{int}	0.085	0.059
$(\sin \theta/\lambda)_{max}$ (Å^{-1})	0.499	0.623
$R[F^2 > 2\sigma(F^2)]$, $wR(F^2)$, S	0.069, 0.173, 1.08	0.110, 0.315, 1.10
Data completeness	0.47	0.31
No. of reflections	545	688
No. of parameters	89	65
No. of restraints	53	53
$\Delta\rho_{max}$, $\Delta\rho_{min}$ (e Å^{-3})	0.29, −0.22	0.58, −0.44

Table S2. Experimental details of the high−pressure collection of polymorph **1β**. For all structures: $C_{18}H_{13}N_4$, M_r = 285.32, monoclinic, $P2_1/c$, Z = 4. Experiments were carried out at 298 K with Mo $K\alpha$ radiation using a Bruker SMART APEX2 area detector. H-atom parameters were constrained.

**Pressure (GPa)**	**0.00**	**0.66**	**0.72**	**1.37**	**1.51**	**2.12**
Crystal #	1	1	2	1	1	1
a, b, c (Å)	19.8508 (16), 3.8614 (4), 19.7823 (16)	19.565 (7), 3.7113 (8), 19.600 (6)	19.479 (12), 3.6660 (11), 19.594 (12)	19.356 (3), 3.5959 (4), 19.521 (4)	19.295 (12), 3.5983 (11), 19.502 (12)	19.164 (3), 3.5215 (4), 19.444 (4)
β (°)	114.729 (3)	114.491 (18)	114.40 (3)	114.331 (10)	114.22 (3)	114.077 (11)
V (Å^3)	1377.3 (2)	1295.1 (7)	1274.3 (12)	1238.0 (4)	1234.8 (11)	1198.0 (4)
μ (mm^{-1})	0.09	0.09	0.09	0.10	0.10	0.10
Crystal size (mm)	0.12 × 0.11 × 0.09	0.12 × 0.11 × 0.09	0.13 × 0.12 × 0.09	0.12 × 0.11 × 0.09	0.12 × 0.11 × 0.09	0.12 × 0.11 × 0.09
Absorption correction ($wR2_{bef}$, $wR2_{aft}$, max : min transmission)	0.0814, 0.0588, 0.8743	0.1001, 0.0508, 0.8421	0.1550, 0.0735, 0.6759	0.1153, 0.0502, 0.8980	0.1658, 0.0879, 0.8331	0.1203, 0.0472, 0.9145
T_{min}, T_{max}	0.652, 0.745	0.628, 0.745	0.504, 0.745	0.669, 0.745	0.621, 0.745	0.682, 0.745
No. of measured, independent and observed [$I > 2\sigma(I)$] reflections	13983, 2822, 1234	5733, 811, 292	5852, 774, 340	5150, 736, 417	6017, 742, 313	4612, 706, 401
R_{int}	0.081	0.151	0.109	0.051	0.150	0.053
$(\sin \theta/\lambda)_{max}$ (Å^{-1})	0.626	0.624	0.595	0.621	0.595	0.623
$R[F^2 > 2\sigma(F^2)]$, $wR(F^2)$, S	0.069, 0.255, 1.01	0.068, 0.215, 1.00	0.071, 0.201, 1.05	0.040, 0.084, 1.11	0.063, 0.168, 1.09	0.039, 0.078, 1.08
No. of reflections	2822	811	774	736	742	706
No. of parameters	200	89	200	199	199	199
No. of restraints	0	53	230	230	230	230
$\Delta\rho_{max}$, $\Delta\rho_{min}$ (e Å^{-3})	0.30, −0.23	0.19, −0.17	0.17, −0.17	0.11, −0.10	0.15, −0.16	0.11, −0.10

Table S2 continued

**Pressure (GPa)**	**2.84**	**3.72**	**4.09**	**4.59**	**5.04**	**6.07**
Crystal #	1	1	1	1	1	1
a, *b*, *c* (Å)	18.950 (4), 3.4374 (4), 19.322 (5)	18.797 (5), 3.3792 (5), 19.274 (5)	18.753 (2), 3.3675 (3), 19.277 (3)	18.634 (7), 3.3280 (7), 19.201 (7)	18.560 (5), 3.3106 (5), 19.165 (6)	18.464 (2), 3.2698 (2), 19.117(3)
β (°)	113.887 (13)	113.647 (14)	113.659 (7)	113.467 (19)	113.414 (14)	113.332(7)
V (Å^3)	1150.9 (4)	1121.4 (5)	1115.0 (2)	1092.2 (6)	1080.6 (5)	1059.8(2)
μ (mm^{-1})	0.10	0.11	0.11	0.11	0.11	0.11
Crystal size (mm)	0.12 × 0.11 × 0.09	0.12 × 0.11 × 0.09	0.12 × 0.11 × 0.09	0.12 × 0.11 × 0.09	0.12 × 0.11 × 0.09	0.12 × 0.11 × 0.09
Absorption correction ($wR2_{bef}$, $wR2_{aft}$, max : min transmission)	0.1526, 0.0691, 0.8776	0.1007, 0.0578, 0.8432	0.1111, 0.0483, 0.8648	0.1472, 0.0754, 0.8344	0.1690, 0.0933, 0.5572	0.1705, 0.0824, 0.7303
Tmin, Tmax	0.654, 0.745	0.629, 0.745	0.645, 0.745	0.622, 0.745	0.415, 0.745	0.544, 0.745
No. of measured, independent and observed [$I > 2\sigma(I)$] reflections	4270, 701, 390	4015, 567, 315	5137, 715, 441	3106, 552, 315	2694, 577, 333	4294, 689, 447
R_{int}	0.053	0.105	0.053	0.077	0.068	0.063
$(\sin \theta/\lambda)_{max}$ (Å^{-1})	0.625	0.595	0.624	0.595	0.594	0.625
$R[F^2 > 2\sigma(F^2)]$, $wR(F^2)$, S	0.050, 0.141, 1.02	0.059, 0.134, 1.19	0.044, 0.098, 1.07	0.043, 0.093, 1.07	0.077, 0.248, 1.08	0.058, 0.153, 1.08
No. of reflections	701	567	715	552	577	689
No. of parameters	199	179	199	174	199	199
No. of restraints	230	167	230	152	230	230
$\Delta\rho_{max}$, $\Delta\rho_{min}$ (e Å^{-3})	0.14, −0.15	0.16, −0.13	0.15, −0.13	0.16, −0.13	0.24, −0.21	0.21, −0.23

Table S2 continued

**Pressure (GPa)**	**6.52**	**6.66**	**7.11**	**7.42**
Crystal #	1	1	1	1
a, b, c (Å)	18.413 (4), 3.2531 (4), 19.094 (5)	18.370 (4), 3.2422 (4), 19.083 (5)	18.346 (3), 3.2314 (4), 19.045 (4)	18.310 (5), 3.2266 (5), 19.037 (6)
β (°)	113.291 (13)	113.214 (12)	113.186 (11)	113.111 (15)
V (Å^3)	1050.5 (4)	1044.5 (4)	1037.9 (3)	1034.4 (4)
μ (mm^{-1})	0.11	0.11	0.11	0.11
Crystal size (mm)	0.12 × 0.11 × 0.09	0.12 × 0.11 × 0.09	0.12 × 0.11 × 0.09	0.12 × 0.11 × 0.09
Absorption correction ($wR2_{bef}$, $wR2_{aft}$, max : min transmission)	0.1610, 0.0819, 0.8031	0.1478, 0.0664, 0.8946	0.1668, 0.0938, 0.7981	0.1069, 0.0531 0.8912
T_{min}, T_{max}	0.599, 0.745	0.667, 0.745	0.595, 0.745	0.664, 0.745
No. of measured, independent and observed [$I > 2\sigma(I)$] reflections	3752, 620, 403	4206, 662, 444	3627, 605, 367	4698, 660, 462
R_{int}	0.060	0.049	0.072	0.049
$(\sin \theta/\lambda)_{\mathrm{max}}$ (Å^{-1})	0.625	0.624	0.625	0.624
$R[F^2 > 2\sigma(F^2)]$, $wR(F^2)$, S	0.043, 0.093, 1.03	0.041, 0.100, 0.99	0.045, 0.121, 1.00	0.043, 0.106, 1.09
No. of reflections	620	662	605	660
No. of parameters	199	199	199	199
No. of restraints	230	230	230	230
$\Delta\rho_{\mathrm{max}}$, $\Delta\rho_{\mathrm{min}}$ (e Å^{-3})	0.15, −0.16	0.13, −0.15	0.17, −0.18	0.13, −0.13
