# Supplementary figures and images for: The Effect of High Pressure on Polymorphs of a Derivative of Blatter’s Radical: Identification of the Structural Signatures of Subtle Phase Transitions
Source: Cryst Growth Des. 2023 Jan 30;23(3):1915–24. doi: 10.1021/acs.cgd.2c01422 (PMC9983015; doi:10.1021/acs.cgd.2c01422)

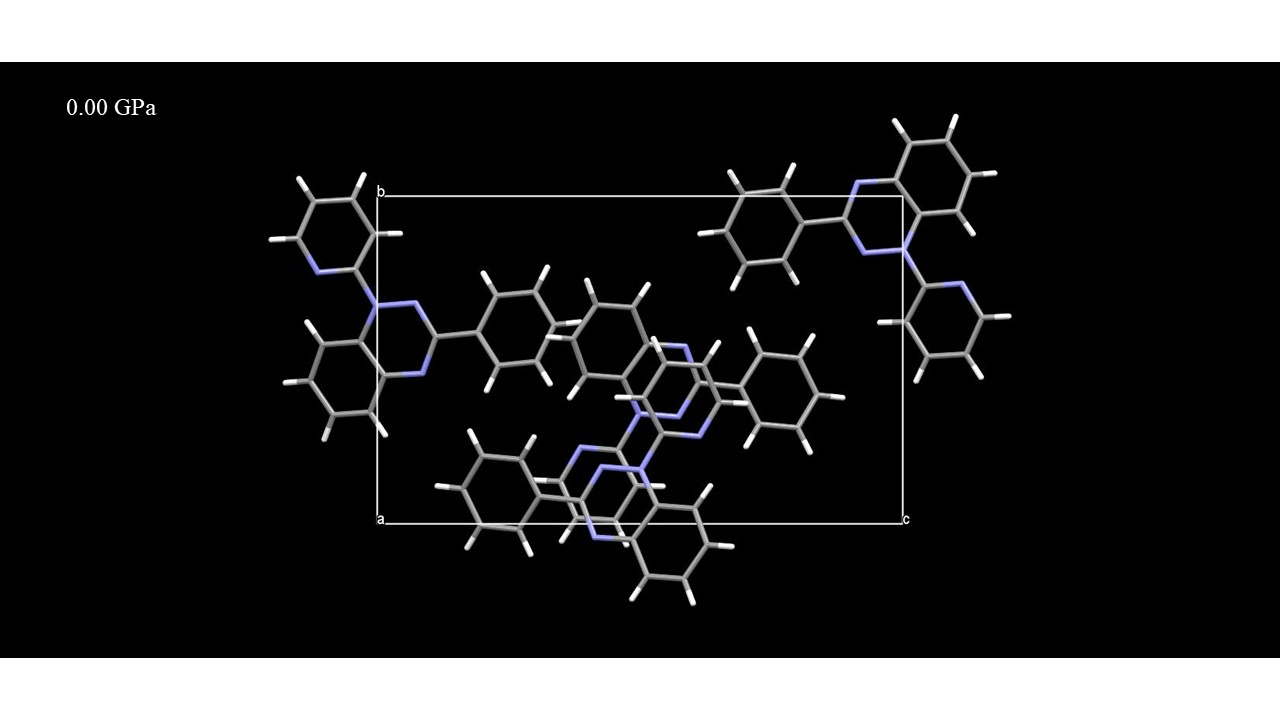

Supplement: Supplementary file 2 — cg2c01422_si_002.zip [file cg2c01422_si_002.zip › Movie1_1alpha_a.gif]

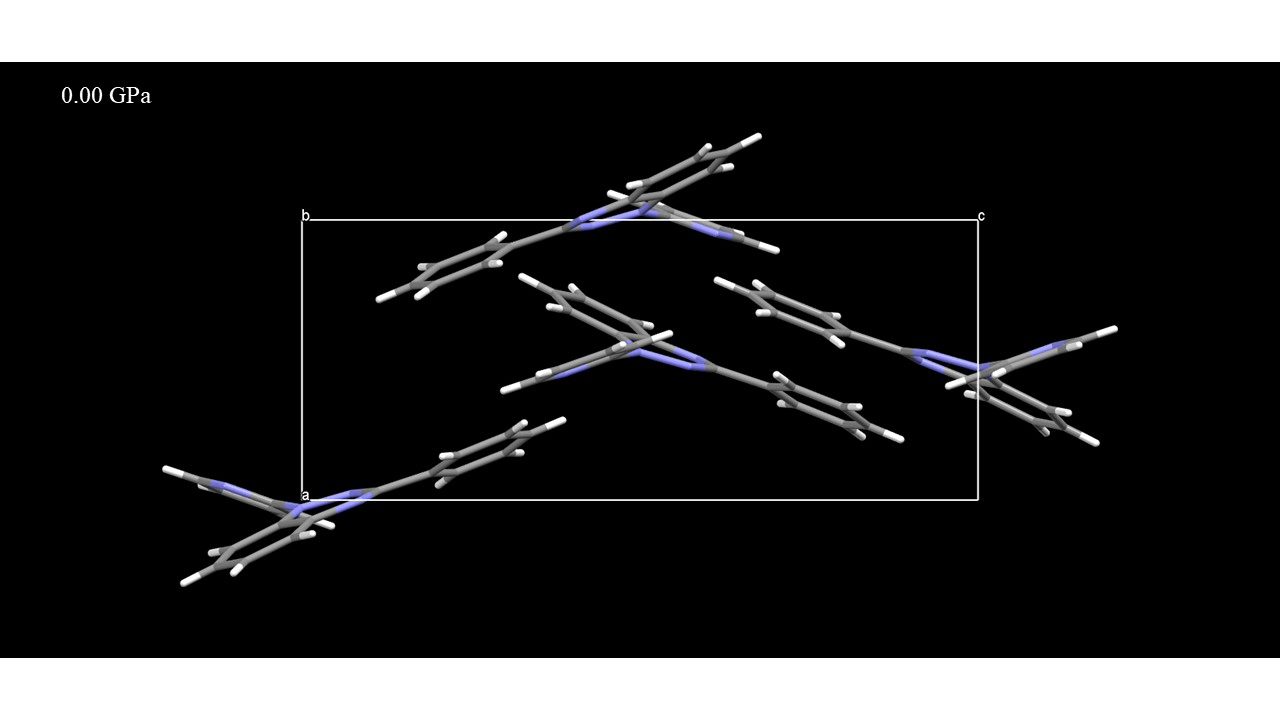

Supplement: Supplementary file 2 — cg2c01422_si_002.zip [file cg2c01422_si_002.zip › Movie2_1alpha_b.gif]

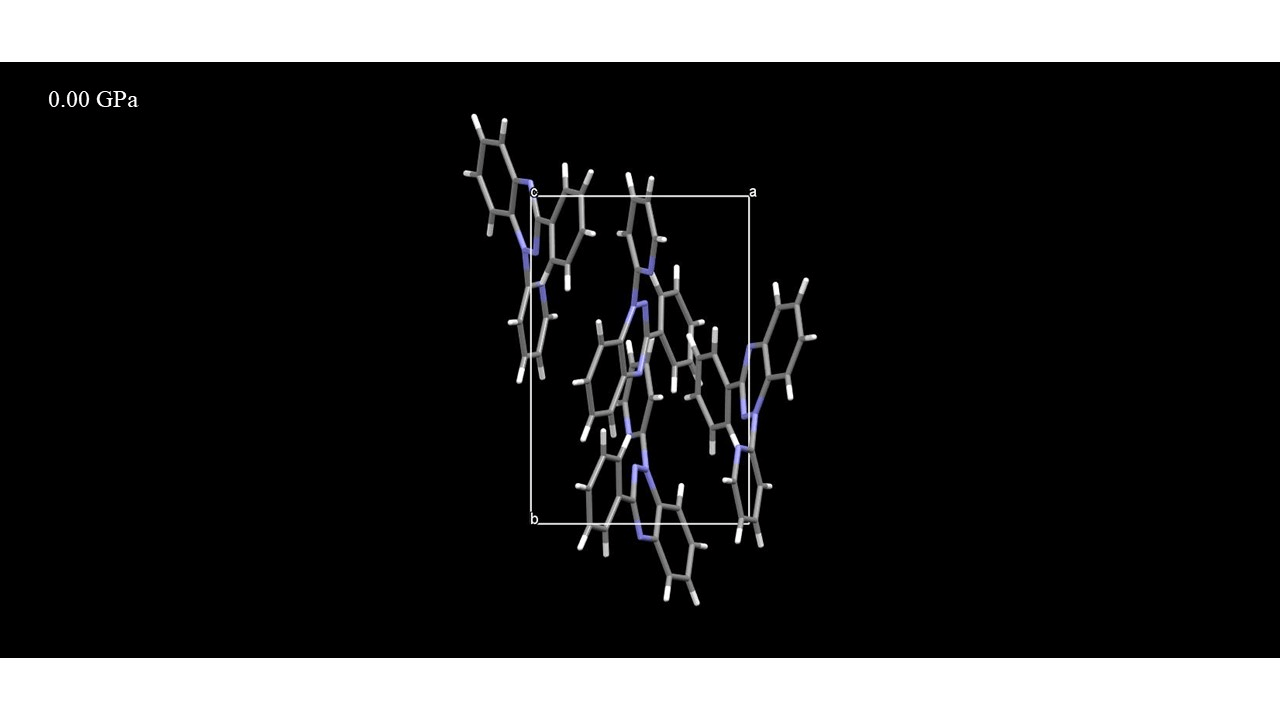

Supplement: Supplementary file 2 — cg2c01422_si_002.zip [file cg2c01422_si_002.zip › Movie3_1alpha_c.gif]

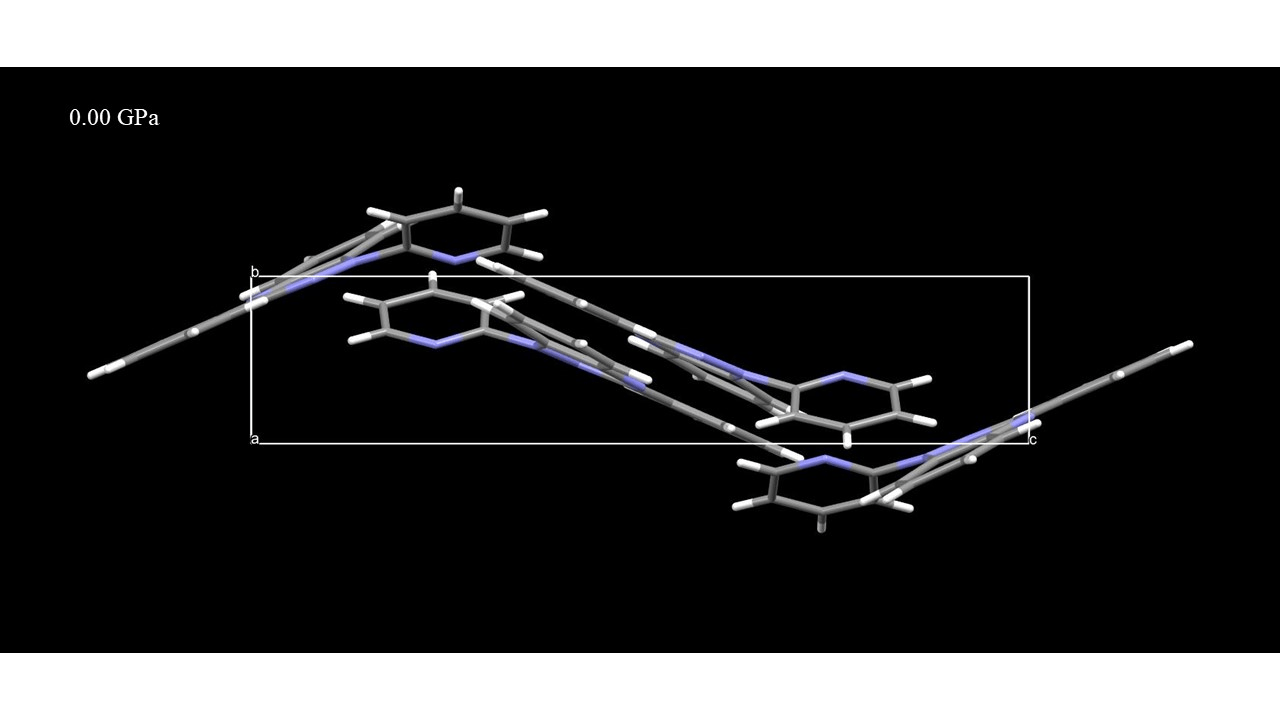

Supplement: Supplementary file 2 — cg2c01422_si_002.zip [file cg2c01422_si_002.zip › Movie4_1beta_a.gif]

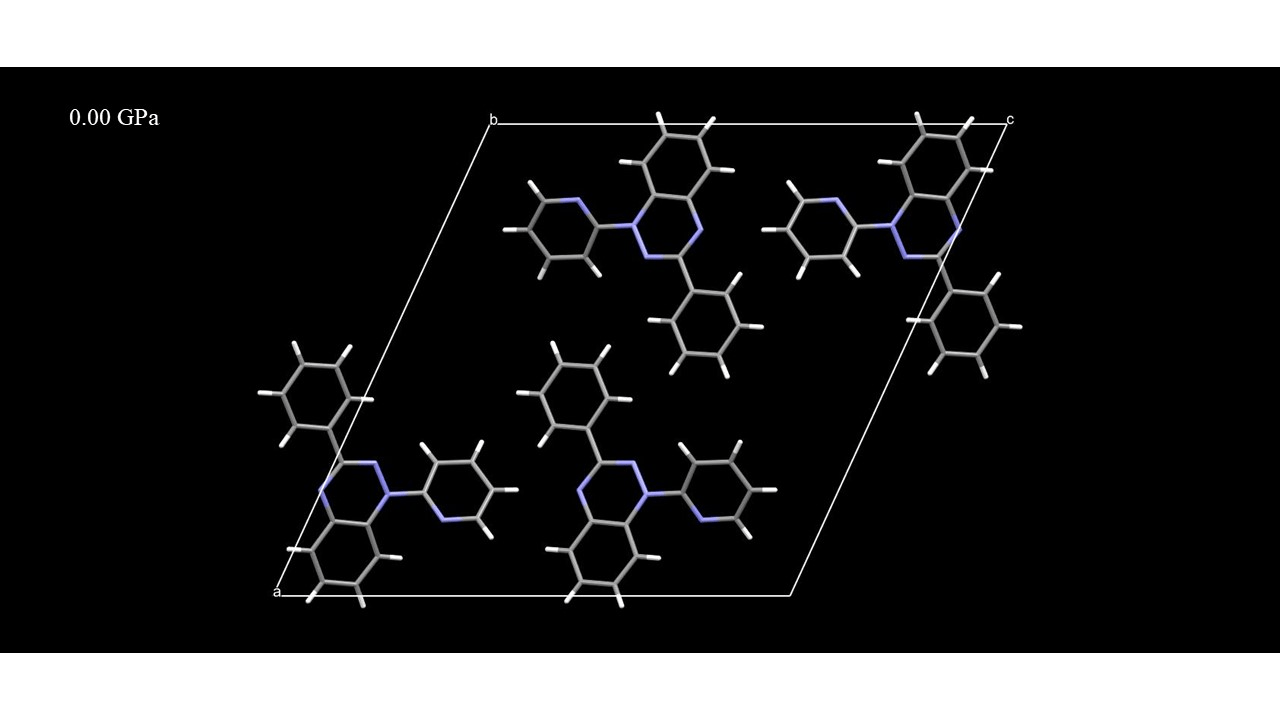

Supplement: Supplementary file 2 — cg2c01422_si_002.zip [file cg2c01422_si_002.zip › Movie5_1beta_b.gif]

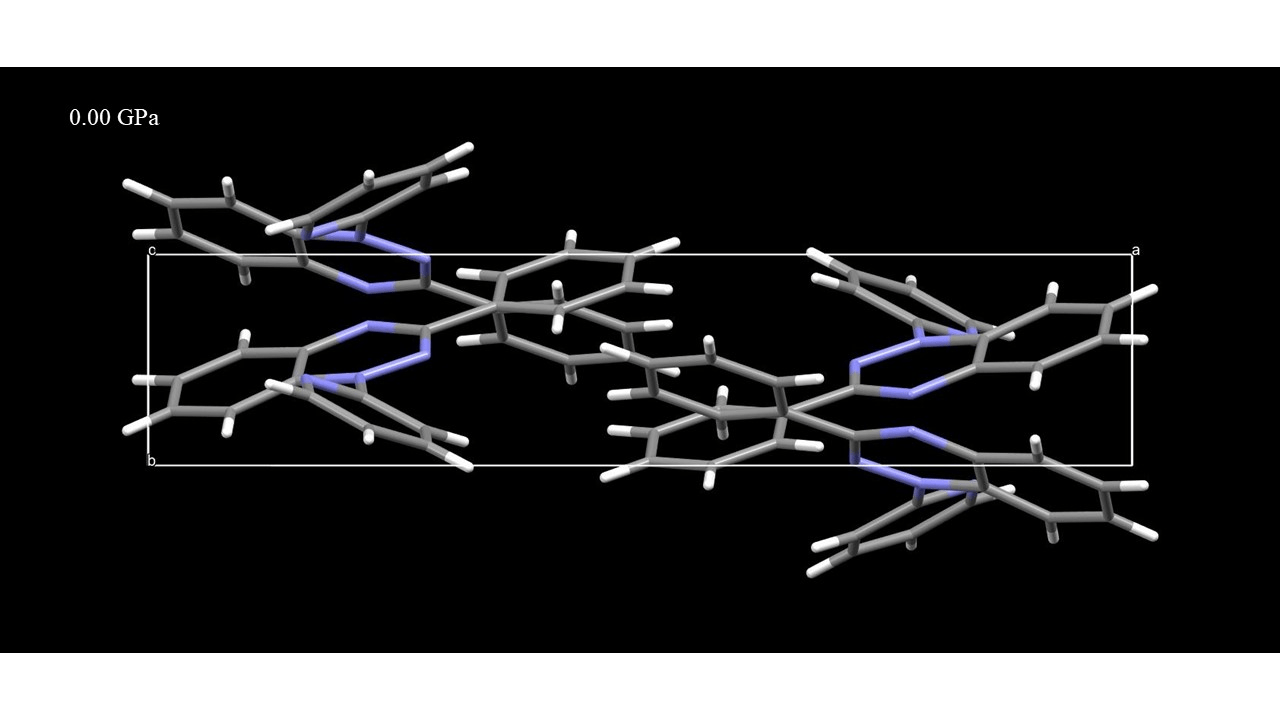

Supplement: Supplementary file 2 — cg2c01422_si_002.zip [file cg2c01422_si_002.zip › Movie6_1beta_c.gif]

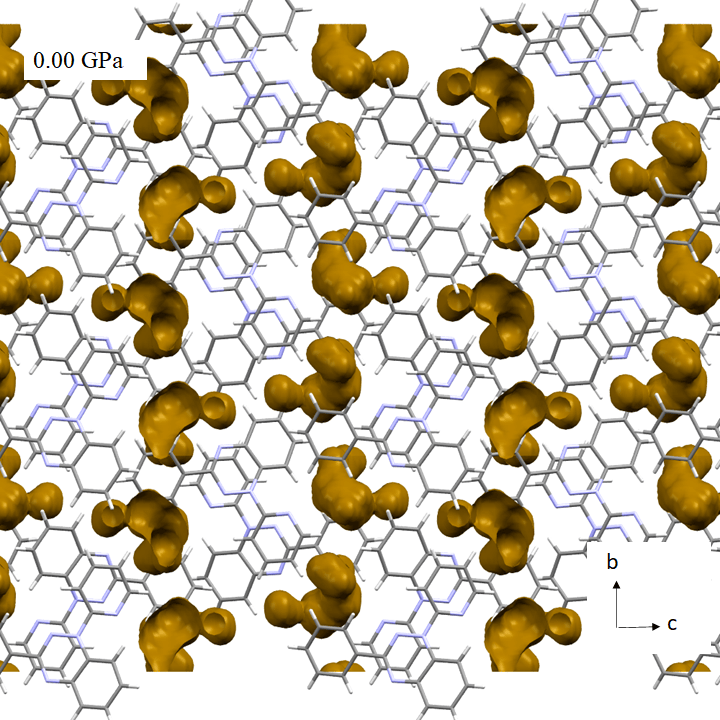

Supplement: Supplementary file 2 — cg2c01422_si_002.zip [file cg2c01422_si_002.zip › Movie7_1alpha_voids.gif]

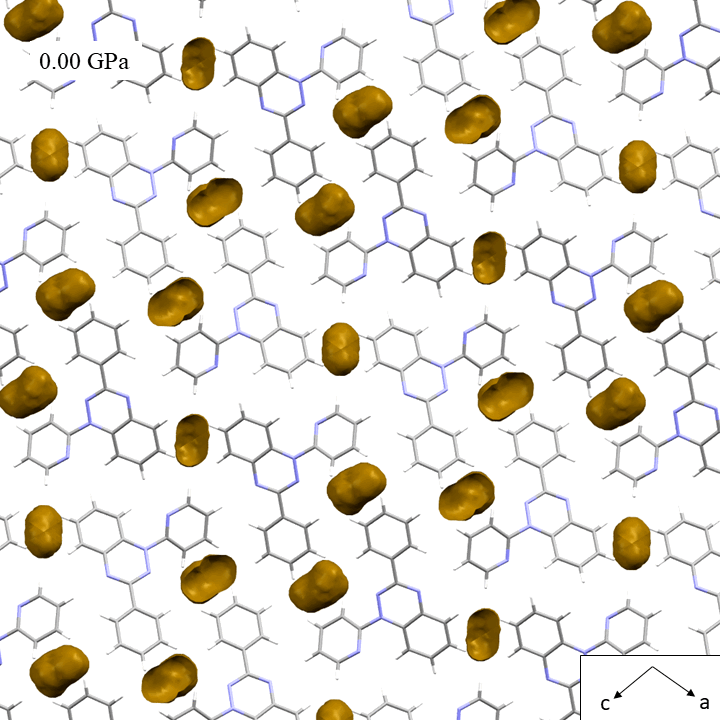

Supplement: Supplementary file 2 — cg2c01422_si_002.zip [file cg2c01422_si_002.zip › Movie8_1beta_voids.gif]
